# Intertumoral heterogeneity impacts oncolytic vesicular stomatitis virus efficacy in mouse pancreatic cancer cells

**DOI:** 10.1128/jvi.01005-23

**Published:** 2023-09-28

**Authors:** Dakota W. Goad, Anna Y. Nesmelova, Laurel R. Yohe, Valery Z. Grdzelishvili

**Affiliations:** 1 Department of Biological Sciences, University of North Carolina at Charlotte, Charlotte, North Carolina, USA; 2 Department of Bioinformatics and Genomics, University of North Carolina at Charlotte, Charlotte, North Carolina, USA; 3 School of Data Science, University of North Carolina at Charlotte, Charlotte, North Carolina, USA; University of Kentucky College of Medicine, Lexington, Kentucky, USA

**Keywords:** vesicular stomatitis virus, oncolytic therapy, pancreatic cancer, pancreatic ductal adenocarcinoma, mouse model of pancreatic cancer

## Abstract

Oncolytic virus (OV) therapy is a promising virus-based approach against various malignancies, including pancreatic ductal adenocarcinoma (PDAC). Our previous studies demonstrated that human PDAC cell lines are highly variable in their permissiveness to OVs. Mouse PDAC cell lines, which are widely used for *in vivo* examination of the adaptive immune responses during OV and other cancer therapies, have never been examined systematically for the impact of intertumoral heterogeneity (the differences observed between tumors in different patients) on OV virus efficacy. Here, we examined phenotypically and genotypically three commonly used allograftable mouse PDAC cell lines (C57BL6 genetic background): Panc02 (derived from chemically induced PDAC; also known as Pan02), and two cell lines originated from PDACs developed in two different KPC (Kras^G12D^, Trp53^R172H^, and PDX-1-Cre) mouse models. Our study (i) characterized the ability of a widely used attenuated oncolytic vesicular stomatitis virus VSV-ΔM51-GFP to infect, replicate in, and kill mouse PDAC cells; (ii) examined their innate antiviral responses; (iii) compared their permissiveness to a non-attenuated VSV-Mwt-GFP and chemotherapeutic drugs; and (iv) analyzed their karyotype and exome. Mouse PDAC cell lines showed high divergence in their permissiveness to VSV-ΔM51-GFP, which negatively correlated with their abilities to mount innate antiviral responses, while all three cell lines were highly permissive to VSV-Mwt-GFP. No correlation was found between resistance to VSV-ΔM51-GFP and chemotherapy. Also, mouse PDAC cell lines showed high divergence in their karyotype and exome. The exome analysis demonstrated that more VSV-ΔM51-GFP-permissive mouse PDAC cell lines harbor mutations in multiple important antiviral genes, such as TYK2, JAK2, and JAK3.

**IMPORTANCE:**

Oncolytic virus (OV) therapy is a promising virus-based approach against various malignancies, including pancreatic ductal adenocarcinoma (PDAC). Our previous studies using various human PDAC cell lines demonstrated that they are highly variable in their permissiveness to OVs. In this study, we examined phenotypically and genotypically three commonly used allograftable mouse PDAC cell lines, which are widely used for *in vivo* examination of the adaptive immune responses during cancer therapies. Mouse PDAC cell lines showed high divergence in their permissiveness to oncolytic vesicular stomatitis virus (VSV), which negatively correlated with their abilities to mount innate antiviral responses. Also, we discovered that more VSV-permissive mouse PDAC cell lines harbor mutations in multiple important antiviral genes, such as TYK2, JAK2, and JAK3. Our study provides essential information about three model mouse PDAC cell lines and proposes a novel platform to study OV-based therapies against different PDACs in immunocompetent mice.

## INTRODUCTION

Pancreatic ductal adenocarcinoma (PDAC) is an aggressive malignancy that accounts for approximately 95% of pancreatic cancers and is the fourth cause of cancer-related deaths in the USA. The 5-year survival rate for PDAC patients has remained around 10%, while survival rates for other cancers have significantly improved (1). The poor survival rate for PDAC is largely attributed to late diagnoses and limited treatment options (2).

Oncolytic virus (OV) therapy is a promising anticancer approach that utilizes replication-competent viruses that preferentially infect, replicate in, and kill cancer cells (3, 4). Vesicular stomatitis virus (VSV) is a nonsegmented negative-strand (NNS) RNA virus (order *Mononegavirales*, family *Rhabdoviridae*, genus *Vesiculovirus*) and a promising OV (5 – 7). VSV-based OVs are already in phases I and II clinical trials (Clinicaltrials.gov trials NCT01628640, NCT03120624, NCT04046445, NCT03865212, NCT03017820, and NCT03647163). VSV can infect and replicate in a wide variety of cell types (8). The pantropism exhibited by VSV is largely due to its use of ubiquitously expressed receptors for attachment and entry into host cells, such as the low-density lipoprotein receptor (9). The oncoselectivity of most OVs, including VSV, is mainly due to defective or suppressed type I interferon (IFN)-mediated antiviral responses in many cancers (10 – 12), because most type I IFN responses are antiproliferative, antiangiogenic, and proapoptotic (13).

Current *in vivo* PDAC mouse model systems fail to recapitulate all key characteristics of human PDAC disease (tumor microenvironment, metastasis, adaptive immune response, etc.) (14 – 16). Importantly, most studies, which use mouse models to investigate PDAC biology and therapies, do not address intertumoral heterogeneity (the differences observed between tumors in different patients) (17). This is an important issue, as our previous studies demonstrated a wide range of permissiveness of human PDAC cells to OVs, from highly permissive to highly resistant, which is largely determined by the abilities of PDAC cells to mount effective innate antiviral responses (18 – 20). Mouse PDAC cell lines, which are widely used for *in vivo* examination of the adaptive immune responses during OV and other therapies, have never been examined systematically for virus-host interactions and the role of intertumoral heterogeneity in OV therapy.

In this study, we examined three different allograftable mouse PDAC cell lines. Two of these cell lines originated from genetically engineered mouse models (GEMMs) of PDAC. GEMMs are created by introducing specific gene mutations in oncogenes and/or tumor suppressor genes that are central in human PDAC, effectively recapitulating PDAC in the mouse. The most robust and well-described PDAC GEMM is the KPC mouse, which is characterized by mutations in the Kras and Trp53, both of which are driven by a pancreas-specific Cre recombinase (via the Pdx1 promoter) which is expressed in all cells of the pancreas from early stages in development (21). Importantly, the KPC GEMM recapitulates many of the PDAC disease features of the human disease, as well as commonly associated disease symptoms such as pain and cachexia (22, 23). Although the resulting PDAC in the KPC GEMM model is highly similar to the human disease, the use of KPC mice is labor intensive, costly to upkeep, and tumor initiation and formation take up to or more than 1 year. Syngeneic mouse models, however, are developed by introducing mouse tumor cells or tissues into immunocompetent mice of the same or similar genetic background by implanting PDAC cells [e.g., KPC cell lines originated from the KPC mouse (24)] or tissue from a C57BL6 background mouse into a “wild-type” (WT) C57BL6 mouse. Syngeneic mouse models can be established in immunocompetent mice either subcutaneously (SC) or orthotopically, and in addition, luciferase can be genetically engineered into the PDAC cell lines, allowing for tumor imaging by measuring the intensity of bioluminescence.

In this study, we examined phenotypically and genotypically three mouse PDAC cell lines (two KPCs and one non-KPC). Our study (i) characterized the ability of a widely used attenuated oncolytic virus VSV-ΔM51-GFP to infect, replicate in, and kill these mouse PDAC cells; (ii) examined their innate antiviral responses; (iii) compared their permissiveness to a non-attenuated VSV-Mwt-GFP and chemotherapeutic drugs; and (iv) analyzed their karyotype and exome. Mouse PDAC cell lines showed high divergence in their permissiveness to VSV-ΔM51-GFP, which negatively correlated with their abilities to mount innate antiviral responses, while all three cell lines were highly permissive to VSV-Mwt-GFP. No correlation was found between resistance to VSV-ΔM51-GFP and chemotherapy. Also, mouse PDAC cell lines showed high divergence in their karyotype and exome. Our study provides essential data about three allograftable model mouse PDAC cell lines and proposes a novel platform to study OV-based therapies against phenotypically different PDACs in immunocompetent mice.

## MATERIALS AND METHODS

### Virus and cell lines

The recombinant virus VSV-ΔM51-GFP was previously described (25), in which the methionine at amino acid position 51 of the matrix protein is deleted and the GFP open reading frame is inserted at position 5 of the viral genome (between VSV G and L genes). Baby hamster kidney fibroblast cells BHK-21 (ATCC CCL-10) were used to grow the virus and to determine viral titers. The recombinant VSV-Mwt-GFP virus was kindly provided by Asit Pattnaik (University of Nebraska). VSV-Mwt-GFP is similar to VSV-ΔM51-GFP but has wt M (26). Titers were determined by adding serial dilutions of the virus to BHK-21 cells using an agarose overlay, followed by calculating either FFU/mL or PFU/mL. To count PFUs, cells were fixed and stained with crystal violet. To count FFUs, VSV-encoded GFP fluorescent foci were quantified using fluorescent microscopy. The mouse PDAC cell lines used in this study were KPC-Luc-4580 (27), KPC-Luc-A (28), and PANC02-Luc (29). The human PDAC cell lines used in this study were SUIT-2 (30), HPAF-II (31), and MIA PaCa-2 (32). The human and mouse origin of all tested PDAC cell lines was confirmed (18) (IDEXX BioAnalytics Case# 18142-2019). KPC-Luc-4580, KPC-Luc-A, MIA PaCa-2, and SUIT-2 cell lines were maintained in Dulbecco’s modified Eagle’s medium [DMEM (Corning, 10-013-CV)]. HPAF-II and BHK cells were maintained in Minimum essential medium Eagle [MEM (Corning, 10-010-CV)]. PANC02-Luc cells were maintained in RPMI 1640 medium (Corning, 10-040-CV). All cell growth media were supplemented with 10% fetal bovine serum [FBS (Gibco)], 4 mM L-glutamine, 900 U/mL penicillin, 900 µg/mL streptomycin, and 1% nonessential amino acids (PANC02-Luc cells were maintained in media without the 1% nonessential amino acids). HPAF-II and BHK cells were additionally supplemented with 17.5% glucose. Cells were kept in a 5% CO_2_ atmosphere at 37°C. For all experiments, cells were kept for no more than 15 passages. All described experiments were approved by the University of North Carolina at Charlotte Institutional Biosafety Committee (IBC).

### Cell growth kinetics

1,000 cells per well in DMEM with 10% FBS were seeded into 96-well plates. Cells were given 24 h to adhere. After each 24-h period, WST-8 (Dojindo, CK04) cell viability reagent was added to each well for 4 h at 37°C in 5% CO_2_, then read using a multiwell plate reader at 450 nm. This was repeated every 24 h for 7 days. Cell doubling time (TD) was calculated using the equation TD  =  (0.693*t*)/ln (Nt/N0) where *t* =  time difference in h during log phase, Nt =  absorbance value at time *t*, and N0 =  absorbance value at the initial time.

### Virus replication kinetics

Virus titers were calculated using standard plaque assays on BHK-21 cells in 12 or 24-well plates. For virus replication kinetics experiments, cells were seeded into 96-well plates and were given 24 h to adhere. Virus dilutions were prepared in DMEM with 0% FBS. Cells were washed with phosphate-buffered saline (PBS), followed by the addition of the virus for 1 h at 37°C. Virus-containing medium was then aspirated and fresh DMEM with 5% FBS was added back to cells and incubated at 37°C in 5% CO_2_ for the duration of the experiment. Virus-encoded GFP fluorescence was measured periodically over a 72-h time course using a fluorescence multiwell plate reader. GFP fluorescence was measured at 485/535 nm.

### Cell viability assay

In a 96-well plate layout, cells were seeded at 90% confluence and were given 24 h to adhere. Cells were then washed once with PBS and mock-infected, or infected at either multiplicity of infection (MOI) 1, 0.1, 0.01, 0.001, or 0.0001. After 1-h incubation at 37°C, the virus was removed and a fresh medium containing 5% FBS was added to each well. At 70 h p.i., WST-8 (Dojindo, CK04) was added to each well for 4 h at 37°C in 5% CO_2_, then read using a multiwell plate reader at 450 nm. Results are expressed as fold change compared with mock treatment.

### Plaque assay

Twelve-well plates were seeded at 90% confluence and were given 24 h to adhere. Cells were infected with VSV-ΔM51-GFP dilutions or mock-infected (control) for 24, 72, and 120 h. One hour after infection, the virus was aspirated and wells were overlaid with 2% agarose (VWR Agarose I-0710) in DMEM with 5% FBS. After 24, 72, or 120 h, formalin was added to fix cells for 4 h. After fixation, agarose was removed and cells were stained with crystal violet (2% crystal violet in methanol). For the plaque assay figures, each row represents one 12-well plate that was cut and stitched to view the highest amount of virus to the least amount of virus from left to right.

### Western blot analysis

Cells were seeded into 12-well plates at 90% confluence and were given 24 h to adhere. The medium was removed and cells were washed once with PBS. Cell lines were either mock-treated or infected with VSV-ΔM51-GFP at MOIs of 0.1 and 0.001 either based on virus titer on BHK-21 cells or based on virus titer on each cell line, in medium with 0% FBS and incubated for 1 h at 37°C. After 1-h incubation, the medium was removed and fresh medium with 0% FBS was added to each well. Cells were lysed and total protein was collected 24 h after infection using buffer as described previously (33). Total protein was separated by electrophoresis on 10% SDS-PAGE gels and electroblotted onto polyvinyl difluoride [PVDF (Millipore IPFL00010)] membranes. Membranes were blocked by using 5% nonfat powdered milk or BSA in TBS-T [0.5 M NaCl, 20 mM Tris (pH 7.5), and 0.1% Tween 20] for at least 1 h at room temperature. Membranes were then incubated in TBS-T with 5% BSA or milk with 0.02% sodium azide and a 1:5,000 dilution of rabbit polyclonal anti-VSV antibodies (raised against VSV virions), a 1:1,000 dilution of rabbit anti-phospho-STAT1 [catalog number 9177S, P-STAT1 (S727) Cell Signaling], a 1:1,000 dilution of rabbit anti-STAT1 (catalog number 14994T, D1K9Y, Cell Signaling), a 1:1,000 dilution of rabbit anti-phospho-STAT2 [catalog number PA5-97361, P-STAT2 (Y690), Invitrogen]. Starbright Blue 700 goat antirabbit (Bio-Rad, 12004161) or antimouse (Bio-Rad, 12004158) IgG fluorescent secondary antibodies at 1:5,000 dilutions were used for fluorescent western blotting detection using the Chemidoc MP imaging system from Bio-Rad. To verify the total protein in each sample (loading control), membranes were stained with Coomassie brilliant blue.

### Cytokine array

Cells were seeded into 12-well plates at 90% confluence and were given 24 h to adhere. The medium was removed and washed once with PBS. VSV-ΔM51-GFP was added at MOIs of 1 and 0.1 (based on each cell line) in a medium with 0% FBS and incubated for 1 h at 37°C. After 1-h incubation, fresh medium was added to each well containing 0% FBS. After 24 h, cell supernatants (for cytokine array) and lysates (for western blot) were collected and stored at −80°C. Supernatants were then sent to EVE Technologies Corp for Mouse Cytokine/Chemokine 44-Plex Discovery Assay Array (MD44).

### IFN sensitivity assay and IFN IC_50_

Cells were seeded into 96-well plates at 90% confluence and were given 24 h to adhere. The medium was removed and cells were washed once with PBS. The virus was then added at MOI of 0.01 in medium with 0% FBS and incubated for 1 h at 37°C. After 1-h incubation, the medium was removed and fresh medium (0% FBS) containing mouse IFN alpha (Invitrogen, 14-8312-80) was added at 2,500, 500, 100, 20, 4, or 0 units/mL. Virus-encoded GFP fluorescence was measured at 485/535 nm periodically over a 73-h time course using a fluorescence multiwell plate reader. IC_50_ values were calculated using GraphPad Prism 9.3.1.

### VSV-ΔM51-GFP versus VSV-Mwt-GFP plaque sizes

Six-well plates were seeded at 90% confluence and were given 24 h to adhere. Cells were infected with sixfold serial dilutions of VSV-ΔM51-GFP or VSV-Mwt-GFP from 1E−4 to 7.7E−8 or mock-infected for 72 h. One hour after infection, the virus was aspirated and wells were overlaid with 2% agarose (VWR Agarose I- 0710) in DMEM with 5% FBS. After 72 h, formalin was added to fix cells for 3 h. After fixation, agarose was removed and cells were stained with crystal violet (2% crystal violet in methanol).

### Relative titer comparison between VSV-ΔM51-GFP and VSV-Mwt-GFP

Twelve-well plates were seeded at 90% confluence and were given 24 h to adhere. Cells were infected with threefold serial dilutions of VSV-ΔM51-GFP or VSV-Mwt-GFP from 1E−4 to 2E−9 or mock-infected for 72 h. One hour after infection, the virus was aspirated and wells were overlaid with 2% agarose (VWR Agarose I-0710) in DMEM with 5% FBS. After 24 h, virus-driven GFP focus forming units (FFUs) were counted using a fluorescent microscope. The results shown are relative to the titer on the KPC-Luc-4580 cell line.

### Comparison of VSV attachment and replication in KPC-Luc-A, KPC-Luc-4580, and PANC02-Luc cells

Cells were seeded into 12-well plates at 95% co0nfluence and were given 24 h to adhere. The medium was removed, and cells were washed with PBS. Cells were placed on ice approximately 5 min before virus infection to cool cells. Virus in DMEM with 0% FBS was added to cells on ice, and cells were incubated for 1 h at 4°C. After incubation, the virus-containing medium was aspirated, and cells were washed three times with cold PBS to remove any unbound virus. Samples were then either collected for cell lysates immediately (to examine attachment) or incubated for an additional 7 h at 37°C (to examine replication) and then cell lysates were collected. Total VSV protein was analyzed by western blot as described above. Membranes were blocked in 5% nonfat milk in TBS-T. Membranes were then incubated with a 1:5,000 dilution of rabbit polyclonal anti-VSV antibodies (raised against VSV virions) in TBS-T with 5% nonfat milk, followed by a 1:10,000 dilution of antirabbit secondary antibodies. To verify the total protein in each sample, loaded membranes were stained with Coomassie blue.

### Gemcitabine and 5-FU IC_50_

For gemcitabine and 5-FU determination, cells were seeded into 96-well plates for approximately 50% confluency in a medium supplemented with 10% FBS. The next day, cells were treated with serial dilutions of either gemcitabine (Selleckchem, S1714) or 5-FU (Selleckchem, S1209). The serial dilution ranges for gemcitabine and 5-FU were 1,000–0.008 µM and 300–0.006 µM, respectively. A WST-8 (Dojindo, CK04) cell viability assay was performed 72 h later. IC_50_ values were calculated using GraphPad Prism 9.3.1.

### Spectral karyotyping and multicolor fluorescence in situ hybridization

Each cell line was grown to 70% confluence in a T-75 flask (Greiner #658170) and sent to the CytoGenomics Core Laboratory at the University of Minnesota. Adherent cells were harvested with colcemid arrest, treated with 0.75 M KCl hypotonic solution, and fixation with 3:1 methanol:acetic acid. The resulting cells were spread onto glass slides according to standard cytogenetic protocols. A spectral karyotyping (SKY) slide was processed according to the manufacturer’s protocol [Applied Spectral Imaging (ASI)]. SKY uses a unique combination of five fluorescent dyes to paint all 24 chromosomes. Seven metaphase cells per sample were examined by SKY using the Olympus BX61 microscope with DAPI and SKY fluorescence filter sets. G-band and SKY metaphase cells were imaged and karyotyped using ASI software. The cytogenetic analyses were performed in the Cytogenomics Shared Resource at the University of Minnesota with support from the comprehensive Masonic Cancer Center NIH Grant #P30 CA077598.

### Exome sequencing and sequence analysis

Each cell line was grown to 90% confluence (about 10^6^ cells), trypsinized, pelleted, frozen, and sent to Azenta US (South Plainfield, NJ, USA) for DNA extraction, library preparations, sequencing reactions, and bioinformatic analysis. DNA was extracted using the PureLink Genomic DNA Mini Kit (Thermo Fisher Scientific) following the manufacturer’s instructions. Genomic DNA samples were quantified using Qubit 2.0 Fluorometer (Thermo Fisher Scientific). Enrichment probes were designed against the region of interest and synthesized through Twist Biosciences—Twist Mouse Comprehensive Panel (South San Francisco, CA, USA). Library preparation was performed according to the manufacturer’s guidelines. Briefly, the genomic DNA was fragmented by acoustic shearing with a Covaris S220 instrument. Fragmented DNA was cleaned up, end-repaired, and adenylated at the 3′ends. Adapters were ligated to the DNA fragments, and adapter-ligated DNA fragments were enriched with limited cycle PCR. Adapter-ligated DNA fragments were validated using Agilent TapeStation (Agilent Technologies) and quantified using Qubit 2.0 Fluorometer. Adapter-ligated DNA fragments were hybridized with biotinylated baits. The hybrid DNAs were captured by streptavidin-coated binding beads. After washing, the captured DNAs were amplified and indexed with Illumina indexing primers. Post-captured DNA libraries were validated using Agilent TapeStation (Agilent) and quantified using Qubit 2.0 Fluorometer and Real-Time PCR (KAPA Biosystems). Libraries were sequenced 2 × 150 on an Illumina HiSeq instrument. For data analysis, sequencing adapters and low-quality bases in raw reads were trimmed using Trimmomatic 0.39. The reads were then aligned to the GRCm37 reference genome using Sentieon 202112.01. Aligned sequences were then sorted and PCR/Optical duplicates were marked, producing BAM files. Somatic SNVs and small INDELs were called by using Sentieon 202112 (TNSeq algorithm). The VCF files generated by the pipeline were then normalized (left alignment of INDELs and splitting multiallelic sites into multiple sites) using bcftools 1.13. Overlapped transcripts were identified for each variant and the effects of the variants on the transcripts were predicted by Ensembl Variant Effect Predictor v104. The most severe impact was selected for each variant and they are used for downstream cohort analysis. The impact of the variants was also classified based on MAF document specifications. The exome data were deposited to the NCBI GenBank Sequence Read Archive (SRA) database under BioProject PRJNA944630. Exome analysis of three murine pancreatic cancer cell lines was deposited under the following accessions: PANC02-Luc (BioSample: SAMN33752692; SRA: SRX21263405), KPC-Luc-4580 (BioSample: SAMN33752691; SRA: SRX21263404), and KPC-Luc-A (BioSample: SAMN33752690; SRA: SRX21263403).

### Statistical analysis

All statistical analyses (except for the exome sequence analysis that is described above) were performed using GraphPad Prism 9.3.1 software. Tests used are indicated in the legends of the figures.

## RESULTS

### High variability in permissiveness of model mouse pancreatic cancer cells to oncolytic vesicular stomatitis virus

**Fig 1:**
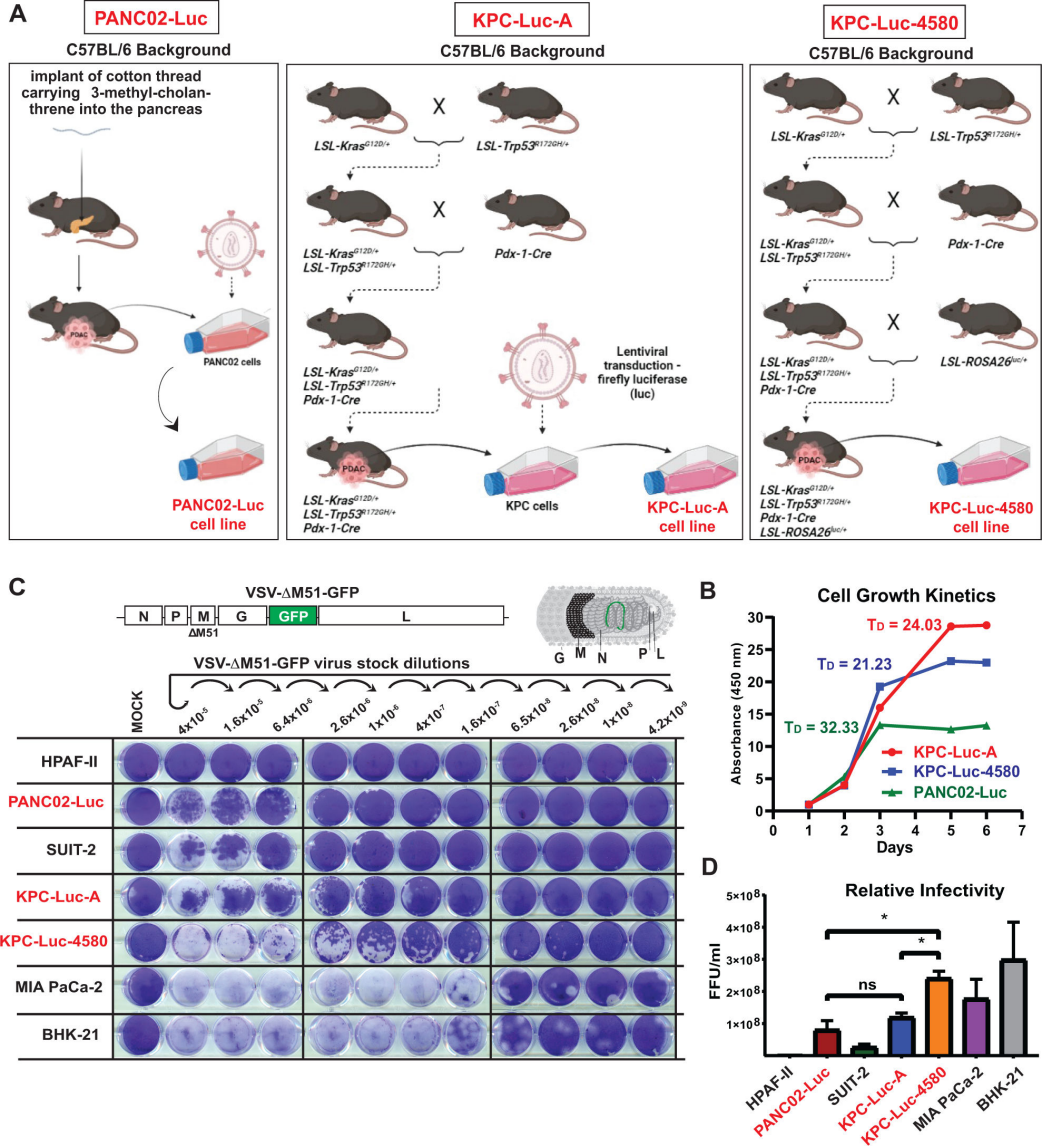
Development and characterization of mouse PDAC cell lines. (**A**) Mouse PDAC cell lines PANC02-Luc, KPC-Luc-A, and KPC-Luc-4580 were previously isolated from PDAC tumors formed in C57BL/6 mice, using different illustrated approaches. (**B**) Cell growth kinetics. 1000 cells were seeded in 96-well plates and growth was measured using WST-8 cell viability assay. The data points and error bars shown represent the means of 16 replicates and SEM of the means, respectively (error bars are too small to be seen). *T*_*D*_ = doubling time. (**C and D**) Relative infectivity to VSV in different cell lines under agarose overlay using a standard PFU assay at 48 h p.i. (**C**) or an FFU assay (**D**), where cell monolayers were examined using fluorescent microscopy to count the virus-di­rected GFP focus forming units (FFUs) at 24 h p.i. to calculate cell line specific FFU/mL for the same VSV-ΔM51-GFP stock. Results shown are representative of five independent experiments. The data points and error bars represent the means and SEM of the means, respectively. Results were analyzed to determine significance using the Student’s *t* test. *, *P* < 0.05.

In this study, we examined three clinically relevant allograftable mouse PDAC cell lines: PANC02-Luc, KPC-Luc-A, and KPC-Luc-4580. These cell lines originated from C57BL6 mice that had three different genetic backgrounds in the pancreas. The PANC02 cell line (alternatively called Pan02 in some publications) is one of the most widely used mouse PDAC cell lines, and it was generated by chemically inducing PDAC in mice via the implantation of 3-methyl-cholantrene (3-MC) (a carcinogen) into the pancreas of genetically unaltered C57BL6 mice (29) (Fig. 1A). The luciferase reporter expressing PANC02-Luc cell line was later generated using lentiviral transduction (34). The KPC-Luc-A cell line was derived from a tumor that developed in an LSL-KrasG12D/+; LSL-Trp53R172H/+; PDX-1-Cre/+ mouse (21), and then the luciferase reporter was inserted using lentiviral transduction (28) (Fig. 1A). The KPC-Luc-4580 cell line (also known as KPC-4580P) was derived from a tumor that developed in an LSL-KrasG12D/+; LSL-Trp53R172H/+; PDX-1-Cre/+, LSL-ROSA26Luc/+ mouse (35) (Fig. 1A). Importantly, the expression of LSL-KrasG12D/+; LSL-Trp53R172H/+ alleles are specific to the pancreas only, as Cre recombinase is driven by the pancreas-specific PDX1 promotor. All three cell lines, PANC02-Luc (34, 36, 37), KPC-Luc-A (28, 38, 39), and KPC-Luc-4580 (35, 40), have been widely used as models to study PDAC biology and treatments for PDAC *in vivo*.

First, we compared growth kinetics and determined the doubling time (*T*_*D*_) of these three cell lines (Fig. 1B). Cells were seeded and cell viability was measured over the course of 6 days. Interestingly, we found that PANC02-Luc cells displayed the slowest overall growth with a *T*_*D*_ of 32.33 days. As mentioned above, the PANC02-Luc cell line was generated in a different way than the KPC (chemically induced via 3-MC) cell lines, which may contribute to the observed difference in growth kinetics. The KPC-Luc-4580 cells displayed the fasted growth with a doubling time of 21.23 days, and KPC-Luc-A cells were intermittent with a *T*_*D*_ of 24.03 days. Although the KPC cell lines were generated similarly, differences in the overall history of each cell line (environment, mutations other than driver mutations, culturing history, etc.) may contribute to the differences in growth kinetics.

This study focused on the oncolytic recombinant VSV-ΔM51-GFP (25). VSV-ΔM51-GFP has a deletion of the methionine residue at position 51 (ΔM51) in the VSV-encoded matrix (M) protein and green fluorescent protein (GFP) reporter gene inserted into the viral genome (Fig. 1C). The ΔM51 mutation prevents VSV-M from binding to the Rae1-Nup98 mRNA export complex required for cellular mRNA transport and subsequent translation (41). Therefore, VSV-ΔM51-GFP is not able to effectively inhibit antiviral responses in initially infected cells (normal or cancer) by disrupting the transport and translation of cellular mRNAs for antiviral genes, which attenuates its replication in normal cells but not in cancer cells as the latter are typically defective in antiviral responses (7, 8, 42, 43). The GFP reporter gene, inserted at position 5 of the viral genome between the VSV G and L genes, allows for monitoring of virus replication and spread based on VSV replication-driven GFP expression (25).

To examine the ability of VSV-ΔM51-GFP to infect and cause oncolysis of PANC02-Luc, KPC-Luc-A, and KPC-Luc-4580, cells were seeded into 12-well plates and infected with serial dilutions of VSV-ΔM51-GFP then overlaid with agarose (to prevent an indiscriminate virus spreading via the flow of the liquid medium during viral propagation). In addition, BHK-21, a baby hamster kidney cell line, was used as a reference cell line for comparison as it is highly permissive to VSV and widely used for VSV amplification and plaque assays. Also, our previous studies demonstrated that there is a wide range of permissiveness of human PDAC cells to OV therapy, from highly permissive to highly resistant, and the success of OV therapy greatly depends on permissiveness of human PDAC cell lines to OVs (18 – 20, 33). Therefore, we wanted to compare permissiveness of the mouse PDAC cell lines to VSV-ΔM51-GFP to that of three well-characterized human PDAC cell lines routinely used in our lab: highly resistant HPAF-II, intermediately resistant SUIT-2, and highly permissive MIA PaCa-2. Following infection, we performed a standard plaque assay at 48 h p.i. to compare the sizes of virus-induced plaques in the cell monolayers (Fig. 1C). In a separate experiment, cell monolayers were examined using fluorescent microscopy to count the virus-directed GFP FFUs at 24 h p.i. to calculate cell line specific FFU/mL for our VSV-ΔM51-GFP stock (Fig. 1D). Not surprisingly, BHK-21 cells were the most permissive among the four tested cell lines (Fig. 1C and D). Among mouse PDACs, PANC02-Luc cells demonstrated the greatest degree of resistance to VSV infection (lowest FFU/mL) (Fig. 1D), VSV spread (smallest size of plaques), and VSV-mediated cell lysis (limited cell clearing), compared to KPC-Luc-A and KPC-Luc-4580 (Fig. 1C). VSV infection, spread, and cell lysis were greater in all three model mouse cell lines compared to HPAF-II cells, which our lab has shown to be one of the most resistant PDAC cell lines to VSV (18 – 20). The KPC-Luc-4580 cell line demonstrated a greater number of FFU/mL, size of plaques, and cell lysis compared to PANC02-Luc and SUIT2 cells, showing a significantly higher number of FFUs and cell lysis compared to KPC-Luc-A (Fig. 1C and D). The KPC-Luc-A cell line showed a greater size of plaques compared to PANC02-Luc (Fig. 1C); however, the differences in FFU/mL were not statistically significant (Fig. 1D). The observed marked difference in the responsiveness of KPC-Luc-A and KPC-Luc-4580 cell lines to VSV-ΔM51-GFP was interesting, as both KPC cell lines originated from similar GEMMs with KrasG12D/+; LSL-Trp53R172H/+; PDX-1-Cre/+ genotype (although KPC-Luc-4580 were originated from a mouse that had another genetic alternation, LSL-ROSALuc/+).

**Fig 2:**
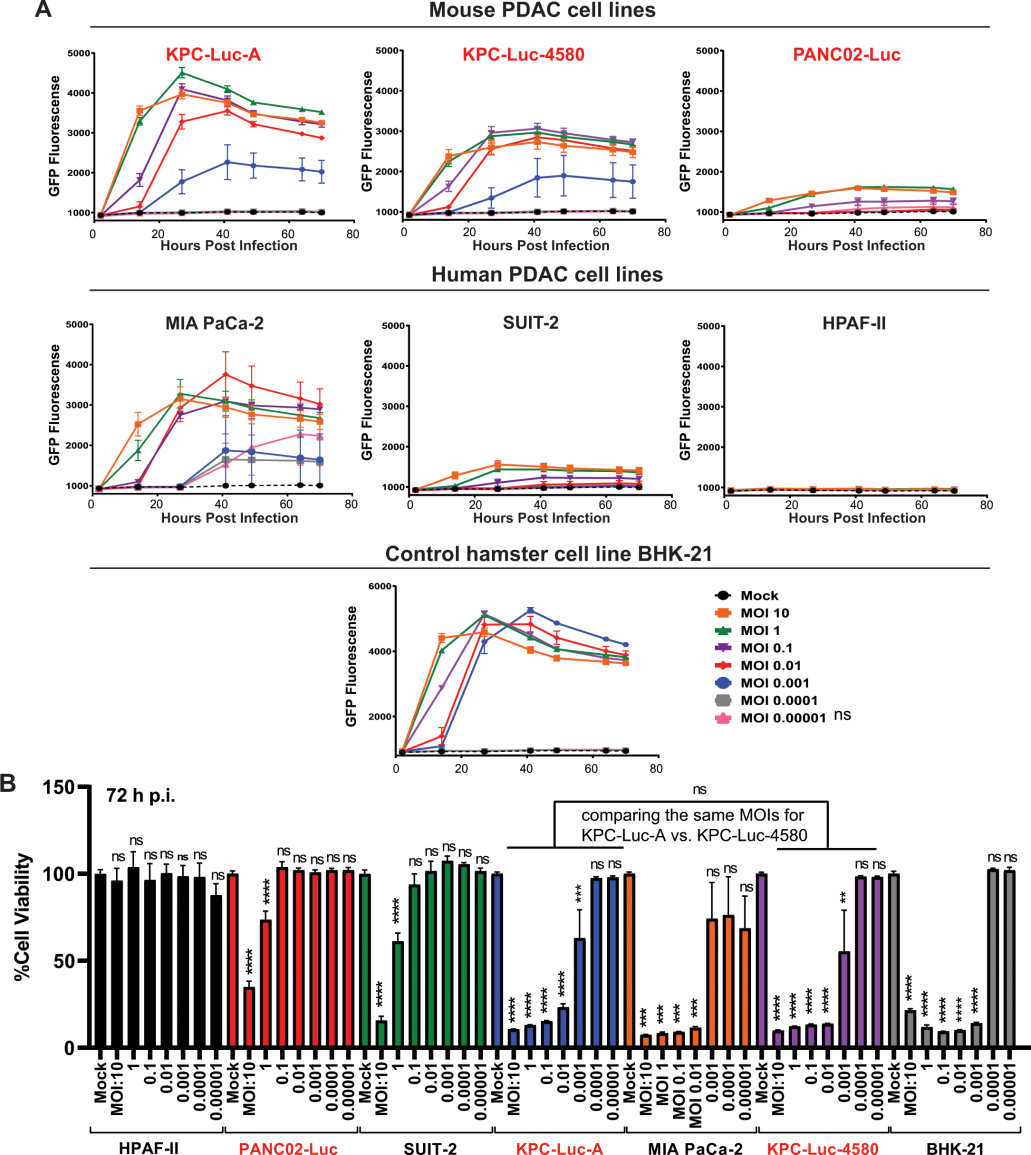
Viral replication kinetics and cell viability. (**A**) Cell lines were either mock-treated or -infected with VSV-ΔM51-GFP at MOIs of 10, 1, 0.1, 0.01, 0.001, 0.0001, or 0.00001 GFP fluorescence was measured over time from 1 to 72 h p.i. (**B**) Cell viability of cells 72 h after infection. Cell lines were either mock-treated or -infected with VSV-ΔM51-GFP at MOIs of 10, 1, 0.1, 0.01, 0.001, 0.0001, or 0.00001. Cell viability was measured at 72 h p.i. using a WST-8 cell viability assay. Results shown are representative of five independent experiments. The data points and error bars shown represent the means and SEM of the means, respectively (some error bars are too small to be seen in the figures). Results were analyzed to determine significance using a one-way ANOVA with a Sidak’s multiple comparison between treatment and mock, and between KPC-Luc-A and KPC-Luc-4580. ns, not significant; **, *P* < 0.01; ***, *P* < 0.001, ****, *P* < 0.0001.

To further examine the ability of VSV to replicate in these mouse PDAC cell lines, we analyzed VSV-driven GFP fluorescence using a panel of human and mouse PDAC cell lines and BHK-21 cells. Cells were seeded in 96-well plates and either mock-infected or infected with VSV-ΔM51-GFP at an MOI of 10, 1, 0.1, 0.01, 0.001, 0.0001, or 0.00001 (based virus titer on BHK-21 cells). As shown in Fig. 2A, very high levels of VSV-driven GFP fluorescence were observed in BHK-21 cells at each tested MOI, which exemplifies that in highly permissive cell lines, VSV replication is good even at extremely low MOIs. In contrast, we observed only small levels of VSV-driven GFP fluorescence in HPAF-II cells, even at the highest MOI tested, which is expected for cells highly resistant to VSV infection. The levels of VSV-driven GFP in each of the mouse PDAC cell lines were lower than those observed in BHK-21 cells but higher than those in HPAF-II. Consistent with Fig. 1, among the three mouse cell lines, the highest levels of VSV-driven GFP fluorescence were observed in KPC-Luc-A and KPC-Luc-4580 cells and the lowest in PANC02-Luc. Interestingly, despite being more resistant to VSV-mediated oncolysis (Fig. 1), KPC-Luc-A showed higher GFP expression levels than KPC-Luc-4580. It should be noted, though, that GFP fluorescence depends not only on VSV replication levels but also on cell viability of virus-infected cells and virus-independent cellular characteristics, such as GFP stability in a given cell line. Therefore, relative GFP fluorescence numbers shown in Fig. 2A should not be used alone to compare permissiveness of KPC-Luc-A and KPC-Luc-4580 cells to VSV. In our previous studies, some highly permissive cell lines, in which VSV was able to replicate to very high levels, displayed only moderately high GFP fluorescence (19, 20).

To examine the effect of VSV infection on cell viability, cells were infected with VSV-ΔM51-GFP at different MOIs (10, 1, 0.1, 0.01, 0.001, 0.0001, or 0.00001) for 70 h, followed by a cell viability assay (Fig. 2B). In general, cell viability negatively correlated with the level of GFP fluorescence at each MOI (Fig. 2A). Of note, some cell lines (such as BHK-21) exhibited low viability even at very low MOI infection. This is likely attributed to the ability of VSV to infect, kill, and spread to neighboring cells even at low MOI due to defective innate antiviral responses and the absence of virus restriction factors. In sharp contrast, we found that in highly resistant HPAF-II cells, cell viability was not affected even at the highest MOIs, which is likely due to the presence of previously demonstrated high levels of VSV restriction factors (such as MX1 and OAS2) in this cell line (18, 19, 44). In most cell lines, we found that cell viability was only affected at several highest MOIs, which is likely due to the limited presence of VSV restriction factors. Among the three mouse PDAC cell lines, PANC02-Luc demonstrated the highest resistance to VSV-mediated reduction in cell viability, followed by KPC-Luc-A and then KPC-Luc-4580. Interestingly, KPC-Luc-A exhibited greater VSV-driven GFP fluorescence compared to KPC-Luc-4580 (Fig. 2A), but this did not correlate with lower cell viability (Fig. 2B).

In general, our data indicate that VSV-ΔM51-GFP differs in its ability to replicate in, spread, and kill each of the three mouse PDAC cell lines. Our data show that PANC02-Luc cells are the overall most resistant to VSV-ΔM51-GFP, followed by KPC-Luc-A and KPC-Luc-4580.

### Resistance of mouse pancreatic cancer cell lines to oncolytic vesicular stomatitis virus is associated with a higher level of antiviral JAK/STAT signaling

**Fig 3:**
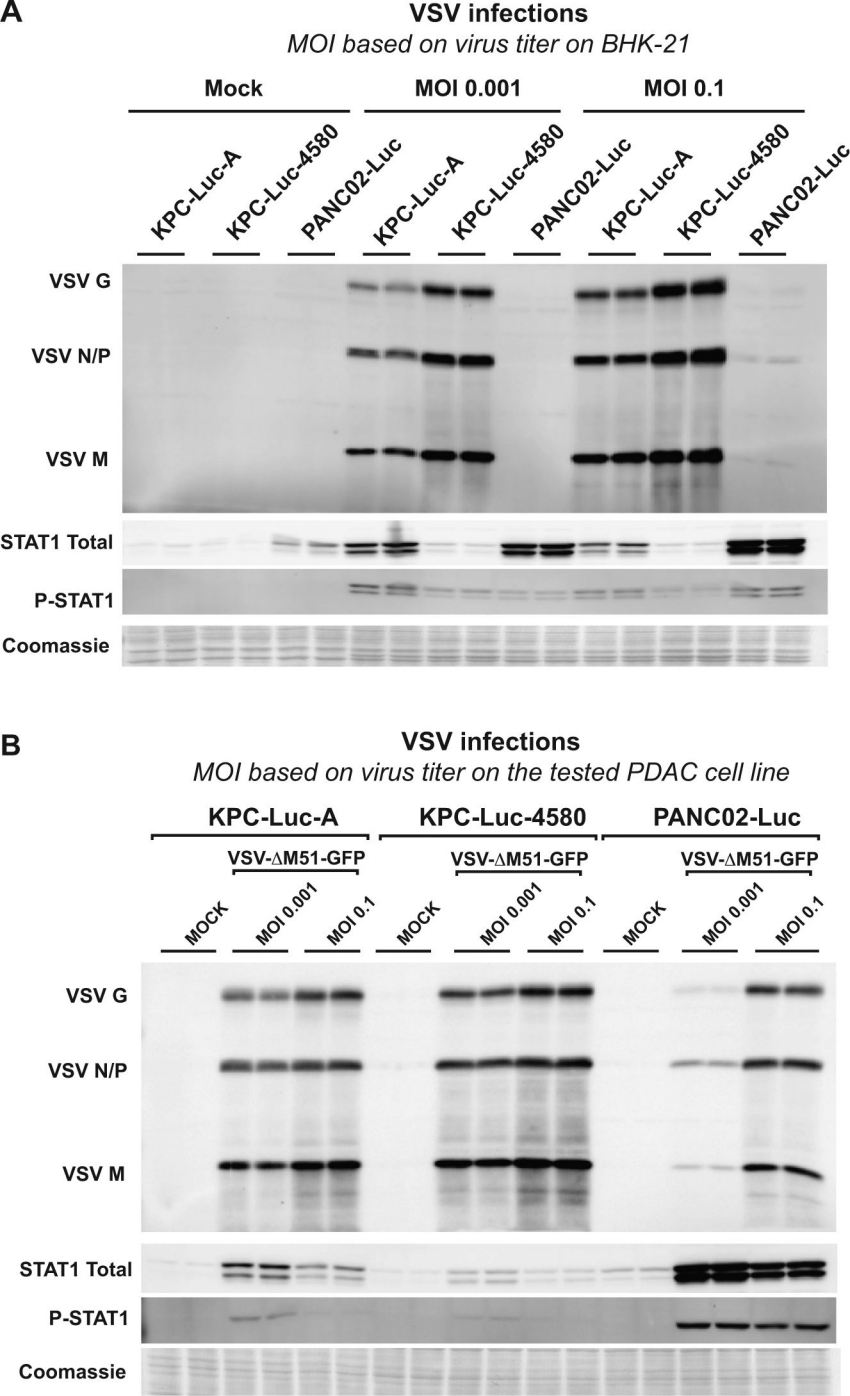
VSV and antiviral protein expression. Cell lines were either mock-treated or -infected with VSV-ΔM51-GFP at MOls of 0.1 and 0.001 either based on virus titer on BHK-21 cells (**A**) or based on virus titer on each cell line (**B**). Protein samples were analyzed at 24 h p.i. by western blotting for expression of VSV proteins (G, N/P, and M), STAT1, and phosphor-STAT1 (P-STAT1). Cell line and treatment conditions are indicated above blots. Equal protein loading is shown by Coomassie blue.

The differences in VSV-ΔM51-GFP replication, spread, and cell lysis observed between the mouse PDAC cell lines suggest potentially varied levels of antiviral signaling. Our previous studies have established that the capacities for type I IFN antiviral signaling between human PDAC cell lines vary dramatically and are strongly correlated with resistance to VSV and other OVs (14, 18 – 20). Here, we hypothesized that more resistant mouse PDAC cell lines have more active type I IFN signaling. To test this hypothesis and examine the role of type I IFN responses in the three mouse PDAC cell lines to the virus, cells were infected with VSV-ΔM51-GFP at MOIs of 0.001 and 0.01 either based on virus titer on BHK-21 cells (Fig. 3A) or based on virus titer on each cell line (Fig. 3B). Total protein was then collected and separated using an SDS-PAGE gel, followed by western blot analysis to determine the expression level of major modulators of type I IFN signaling, total STAT1, and phosphorylated STAT1 (p-STAT1) (Fig. 3). VSV protein expression was also analyzed to assess VSV replication in each cell line. Under both conditions (Fig. 3A and B), we found the greatest level of VSV protein expression in KPC-Luc-4580 cells, an intermediate level in KPC-Luc-A, and the least amount of VSV protein expression in PANC02-Luc cells. Our lab has previously shown that highly resistant human PDAC cell lines exhibit constitutive expression of p-STAT1 with corresponding upregulation of ISGs, even in the absence of VSV infection (18, 19). While no constitutive expression of p-STAT1 was observed in any of the tested mock-treated mouse PDAC cell lines, we found that in VSV-infected samples, PANC02-Luc cells show robust p-STAT1 and total STAT1 expression compared to KPC-Luc-A and KPC-Luc-4580 at both MOIs of 0.001 and 0.1. There was little to no p-STAT1 or total STAT1 expression in KPC-Luc-4580 cells, and there were intermediate expression levels of p-STAT1 and total STAT1 in KPC-Luc-A cells (Fig. 3). In agreement with our hypothesis that more resistant mouse PDAC cell lines would have greater type I IFN signaling, these data show an inverse correlation between total STAT1 and p-STAT1 expression and levels of VSV protein expression. These data suggest that the differences in the ability of VSV-ΔM51-GFP to infect, spread, and kill mouse PDAC cell lines may be due to varied levels of antiviral signaling.

**Fig 4:**
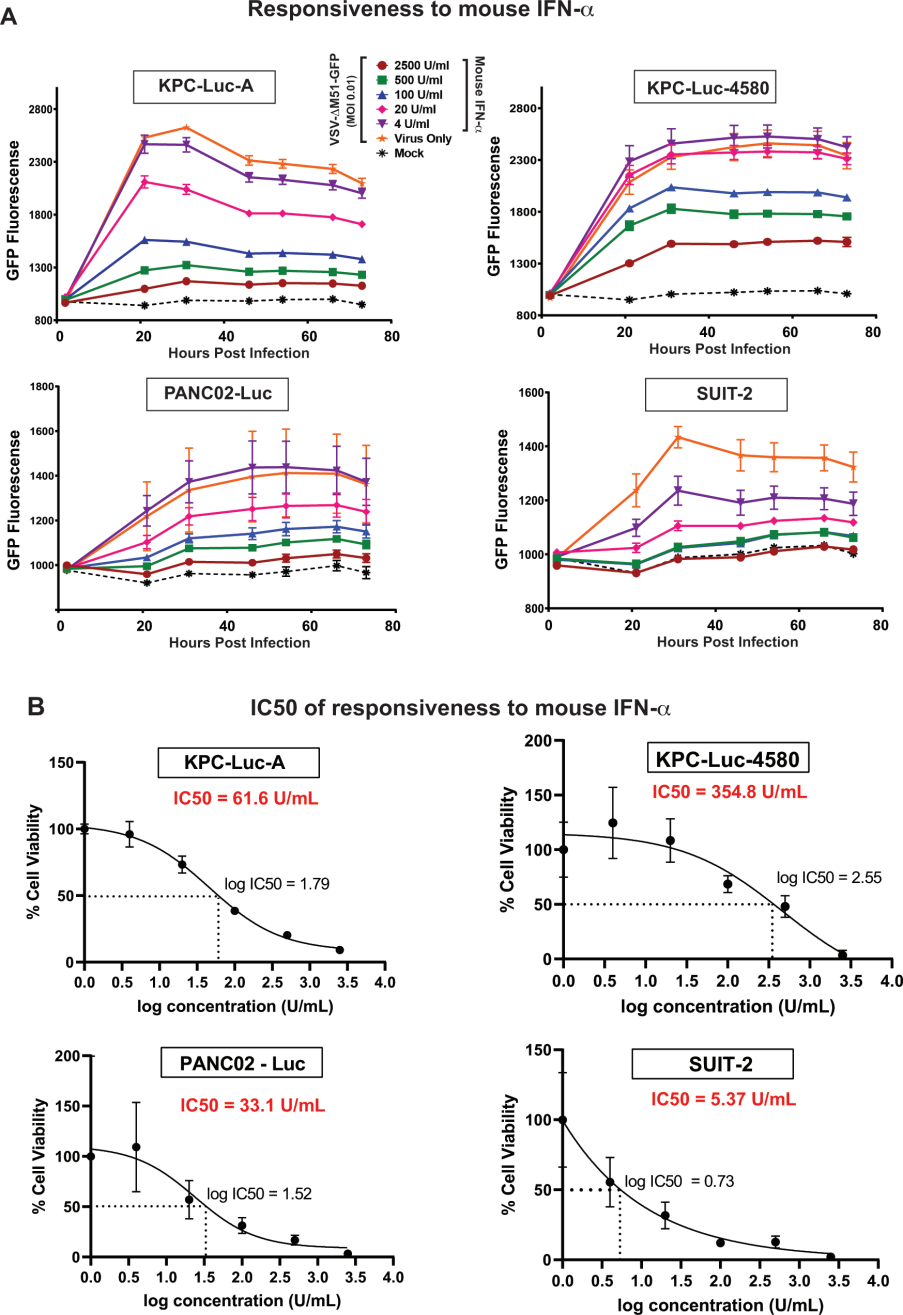
Responsiveness to mouse IFN-α. (**A**) Cells were either mock-treated or -infected with VSV-ΔM51-GFP at an MOI of 0.01 (based on BHK-21 cells). Cells were then treated with mouse IFN-α (mIFN-α) at 2,500, 500, 100, 20, 4, or 0 (virus only) units/mL. GFP fluorescence was measured overtime from 1 to 73 h p.i. (**B**) The half maximal inhibitory concentration (IC_50_) of mIFN-α was determined by using GraphPad Prism 9.3.1. Results shown are representative of three independent experiments. The data points and error bars shown represent the means and SEM of the means, respectively (some error bars are too small to be seen in the figures).

To further study the role of antiviral signaling in mouse PDAC cell lines, we examined the antiviral effect of mouse IFNα (mIFNα) on VSV-ΔM51-GFP replication in each cell line. We also used the reference human PDAC cell line, SUIT-2, as this cell line is extensively used in our lab and exhibits an intermediate level of type I IFN antiviral signaling. Importantly, human cells are known to be also sensitive to mIFNα (45, 46). Our previous studies have demonstrated that, excluding highly permissive PDAC cell lines, most human PDAC cell lines can respond to type I IFNs (20). Therefore, we hypothesized that the more resistant a cell line is to virus infection, the less mIFNα would be needed to inhibit virus replication. Cells were mock-infected or virus-infected with VSV-ΔM51-GFP at MOI of 0.01 (calculated based on VSV titer on BHK-21 cells). Immediately after the virus was added, cells were supplemented with 2,500, 500, 20, or 4 U/mL of mIFNα. VSV-driven GFP expression was measured over the course of 78 h (Fig. 4A). For each cell line, mIFNα inhibited virus replication in a dose-dependent manner, indicating that each cell line has at least some level of intact type I IFN antiviral signaling capability. However, the overall sensitivity of each line to mIFNα was different. To measure it, we calculated the half maximal inhibitory concentration (IC_50_) for mIFNα, the amount of mIFNα needed to result in 50% inhibition of virus replication (inversely correlates to the antiviral signaling capability of the cells) (Fig. 4B). The analysis shows that SUIT-2 cells exhibit the greatest level of antiviral capability (IC_50_ = 5.37 U/mL), followed by PANC02-Luc (IC_50_ = 33.1 U/mL), KPC-Luc-A (IC_50_ = 61.6 U/mL), and KPC 4580 (IC_50_ = 354.8 U/mL). These data support our hypothesis that resistance of mouse KPC cells to VSV is at least in part due to their abilities to mount antiviral responses. In agreement with this hypothesis, we see that PANC02-Luc cells have both the greatest sensitivity to mIFNα and the greatest overall resistance to VSV-ΔM51-GFP among tested mouse PDAC cell lines.

**Fig 5:**
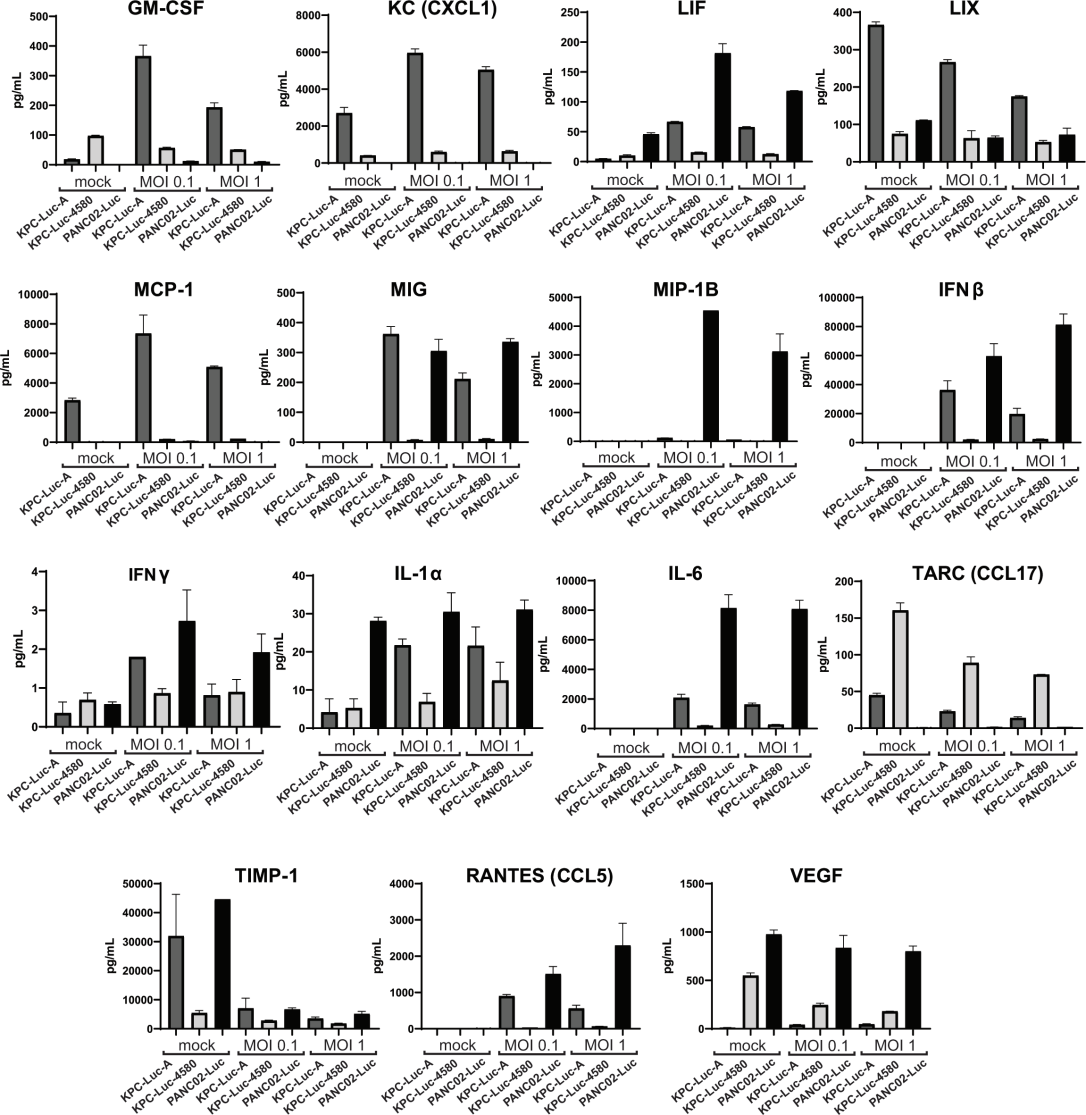
Cytokine array. Cells were either mock-treated or -infected with VSV-ΔM51-GFP at MOIs of 1 and 0.1 (titer was calculated based on each individual cell line). At 24 h p.i., cell supernatants were collected and sent to Eve Technologies for Mouse Cytokine/Chemokine 44-Plex Discovery Assay array (MD44). The data points and error bars shown represent the means and SEM of the means, respectively.

As mentioned above, the functional innate immune responses require not only the ability of cells to respond to IFN but to produce and secrete IFNs and other antiviral cytokines. We hypothesized that cell lines would differ in their ability to produce and secrete such cytokines. To test this hypothesis, cells were either mock-treated or infected with VSV-ΔM51-GFP at MOI 1 and 0.1 (based on virus titer on each cell line). At 24 h p.i., cell supernatants were collected and analyzed using the Mouse Cytokine/Chemokine 44-Plex Discovery Assay Array (MD44), which includes critical antiviral and proinflammatory cytokines known to control viral infections. Out of the 44 different cytokines that were measured, we show in Fig. 5 only 15 of the cytokines that were differentially produced between the mouse PDAC cell lines (the complete analysis is shown in Table S1). In the absence of VSV infection, we found no detectable levels of MIG (C-X-C motif ligand 9), MIP-1 (macrophage inflammatory protein 1β), IFN-β, or IL-6 (interleukin 6) in any of the three mouse cell lines (Fig. 5; Table S1). For all other cytokines, there were detectable levels in at least one of the three cell lines. The levels of basal production of some cytokines in the absence of VSV infection were strikingly different between cell lines, e.g., LIX (C-X-C motif chemokine 5), IL-1α (interleukin 1α), IL-6 (interleukin 6), TARC (C-C motif chemokine ligand 17), and VEGF (vascular endothelial growth factor). PANC02-Luc cells produced the highest levels of IL-1α, IL-6, and VEGF while producing the least amount of TARC. KPC-Luc-A cells produced the highest amount of LIX and the lowest amount of VEGF. KPC-Luc-4580 cells produced the highest amount of TARC while producing the lowest amount of LIX, IL-1α, and IL-6. In the presence of VSV infection, the levels of IFN-β and IFN-γ (type II IFN) were greatest in PANC02-Luc cells, which is consistent with our previous data indicating higher “antiviral status” of this cell line (Fig. 3 and 4). Also, consistent with our previous data, we found that KPC-Luc-A exhibited higher levels of IFN-β and IFN-γ compared to KPC-Luc-4580 (Fig. 5). In general, the cytokine analysis data show a positive correlation between levels of produced IFN-β and IFN-γ in each cell line and the observed degree of resistance to VSV infection. Interestingly, the levels of other cytokines between the three cell lines vary dramatically, even between KPC-Luc-A and KPC-Luc-4580, which have the same tumor driver mutations. For example, the levels of TARC produced in KPC-Luc-4580 cells are highest between cell lines, versus the levels of MCP-1 (monocyte chemoattractant protein-1) that was highest in KPC-Luc-A cells. The levels of produced cytokines in MOI 0.1 versus MOI 1 were relatively similar with only some exceptions, such as GM-CSF (granulocyte-macrophage colony-stimulating factor) and MIG (CXCL9). In general, there is no overall positive correlation between cytokine production and the degree of resistance to VSV-ΔM51-GFP infection between the three mouse PDAC cell lines. However, type I/II IFN cytokine production (e.g., IFNβ and IFNγ) was greatest in PANC02-Luc cells.

### Comparing performance of VSV-ΔM51-GFP and VSV-Mwt-GFP viruses in mouse pancreatic cancer cell lines

Our data suggest that the observed differences in permissiveness of mouse PDAC cell lines to VSV-ΔM51-GFP are likely due to the higher levels of antiviral restriction factors (constitutive and/or induced) present in more resistant cell lines. This hypothesis is consistent with our previous studies demonstrating that the ability of cells to mount functional type I IFN antiviral responses is a major determinant of resistance of human PDAC cell lines to VSV-ΔM51-GFP (19, 20). However, it is still possible that alternatively or in addition to this mechanism, more resistant cell lines have limited levels of some positive factors required for VSV replication. To address the latter hypothesis, we compared the mouse PDAC cell lines for their abilities to support replication of either VSV-ΔM51-GFP or VSV-Mwt-GFP (nonattenuated VSV containing a wild-type matrix (M) protein and GFP reporter gene). As described above, compared to VSV with a wt M gene, VSV-ΔM51-GFP is much more sensitive to type I IFN antiviral responses, as it has a deletion of methionine 51 in the VSV M protein, resulting in the inability of this protein to inhibit nucleus-to-cytoplasm transport of cellular mRNA, including antiviral transcripts. If mouse PDAC cell lines differ in their levels of positive factors (unrelated to immune response) of VSV replication, we expected to observe similar differences in the ability of cell lines to support VSV-Mwt-GFP replication. However, if the major mechanism of resistance is the antiviral potentials of mouse PDAC cell lines, we expected to see smaller differences between cell lines for VSV-Mwt-GFP.

**Fig 6:**
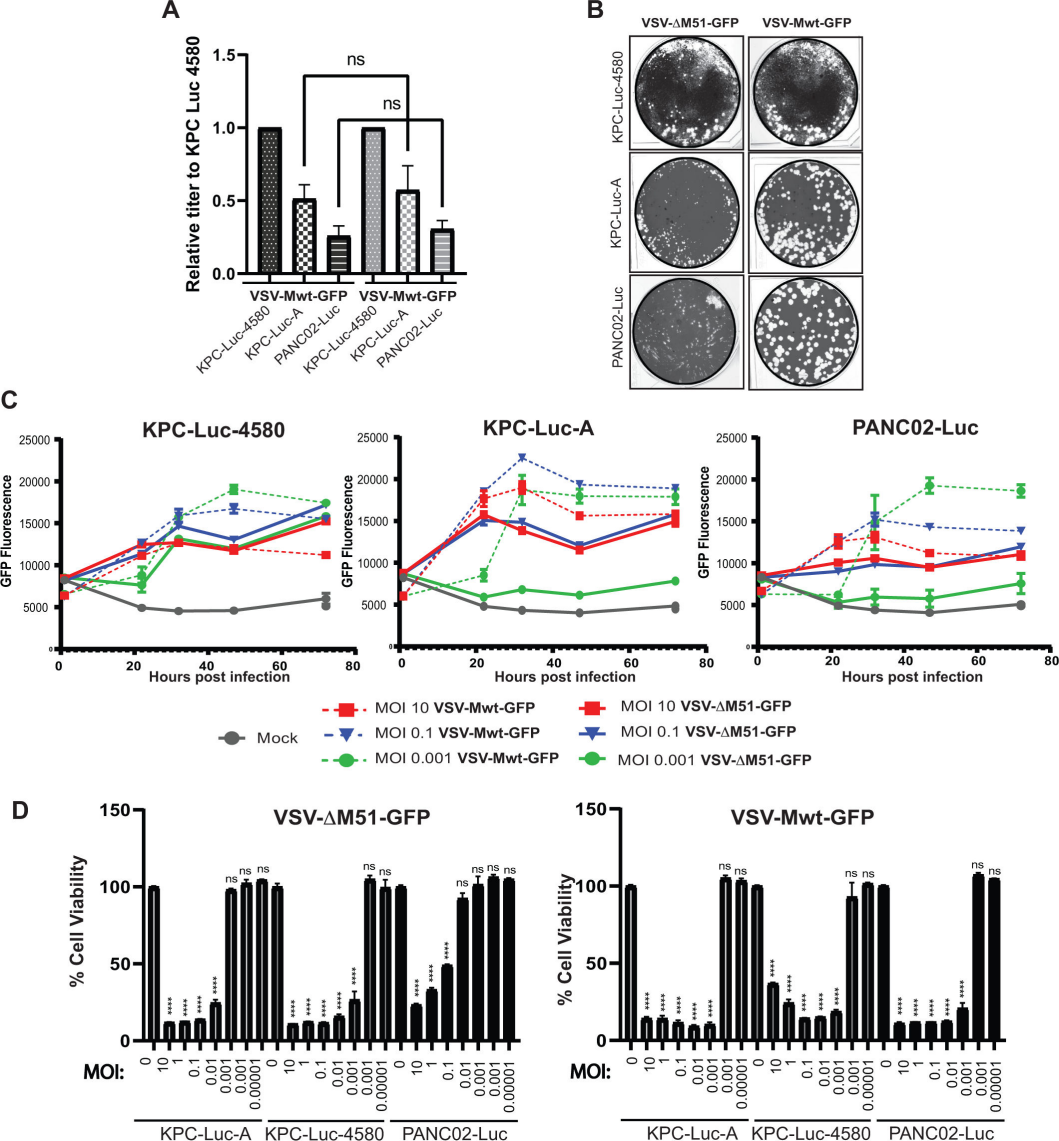
Comparing replication of VSV-ΔM51-GFP and VSV-Mwt-GFP in mouse PDAC cell lines. (**A**) Relative titer comparison between VSV-ΔM51-GFP and VSV-Mwt. (**B**) Relative plaque sizes of VSV-ΔM51-GFP and VSV-Mwt-GFP. Cell lines were infected with serial dilutions of each virus under agarose. Cells were fixed and stained at 72 h p.i. Results shown are representative wells of four independent experiments. (**C**) Viral infection kinetics. Cell lines were either mock-treated or -infected with each virus at different MOls. GFP fluorescence was measured over time from 1 to 72 h p.i. (**B**) Cell viability 72 h after infection. Cell viability was measured at 72 h p.i. using a WST-8 cell viability assay. The data points and error bars shown represent the means and SEM of the means, respectively (some error bars are too small to be seen in the figures). (**D**) Cell viability was measured at 72 h p.i. using a WST-8 cell viability assay. All virus titers were based on BHK-21 cells. Results were analyzed to determine signifi­cance using a one-way ANOVA with a Sidak’s multiple comparison between treatment and mock.****, *P* < 0.0001.

To compare VSV-ΔM51-GFP and VSV-Mwt-GFP, we first tested the abilities of each virus to initiate infections (Fig. 6A) and spread cell-to-cell (Fig. 6B) on each of the mouse PDAC cell lines. Cell lines were infected with serial dilutions of VSV-ΔM51-GFP or VSV-Mwt-GFP under agarose. To determine the abilities of viruses to initiate infection, FFU/mL was calculated for each virus and cell line, and the results shown in Fig. 6A show the ratios of each virus titer to its titer on the KPC-Luc-4580 cell line, which was the most permissive to VSV-ΔM51-GFP. To determine the abilities of viruses to spread, we compared the size of plaques at 72 h p.i. (Fig. 6B). Our data show that VSV-ΔM51-GFP or VSV-Mwt-GFP have similar abilities to initiate infection in mouse PDAC cell lines (Fig. 6A). However, we observed a dramatic increase in the size of plaques for VSV-Mwt-GFP (compared to VSV-ΔM51-GFP) in KPC-Luc-A and especially in PANC02-Luc (Fig. 6B). Also, compared to VSV-ΔM51-GFP, VSV-Mwt-GFP showed much better replication kinetics (Fig. 6C) and stronger inhibition of cell viability (Fig. 6D) in KPC-Luc-A and PANC02-Luc cell lines. Taken together, our data in Fig. 6 support the hypothesis that antiviral signaling is a major determinant of resistance of mouse PDAC cell lines to VSV-ΔM51-GFP, as all three cell lines were highly permissive to VSV-Mwt-GFP replication and VSV-Mwt-GFP-mediated oncolysis.

### Role of the virus attachment in permissiveness of mouse pancreatic cancer cell lines to VSV-ΔM51-GFP

**Fig 7:**
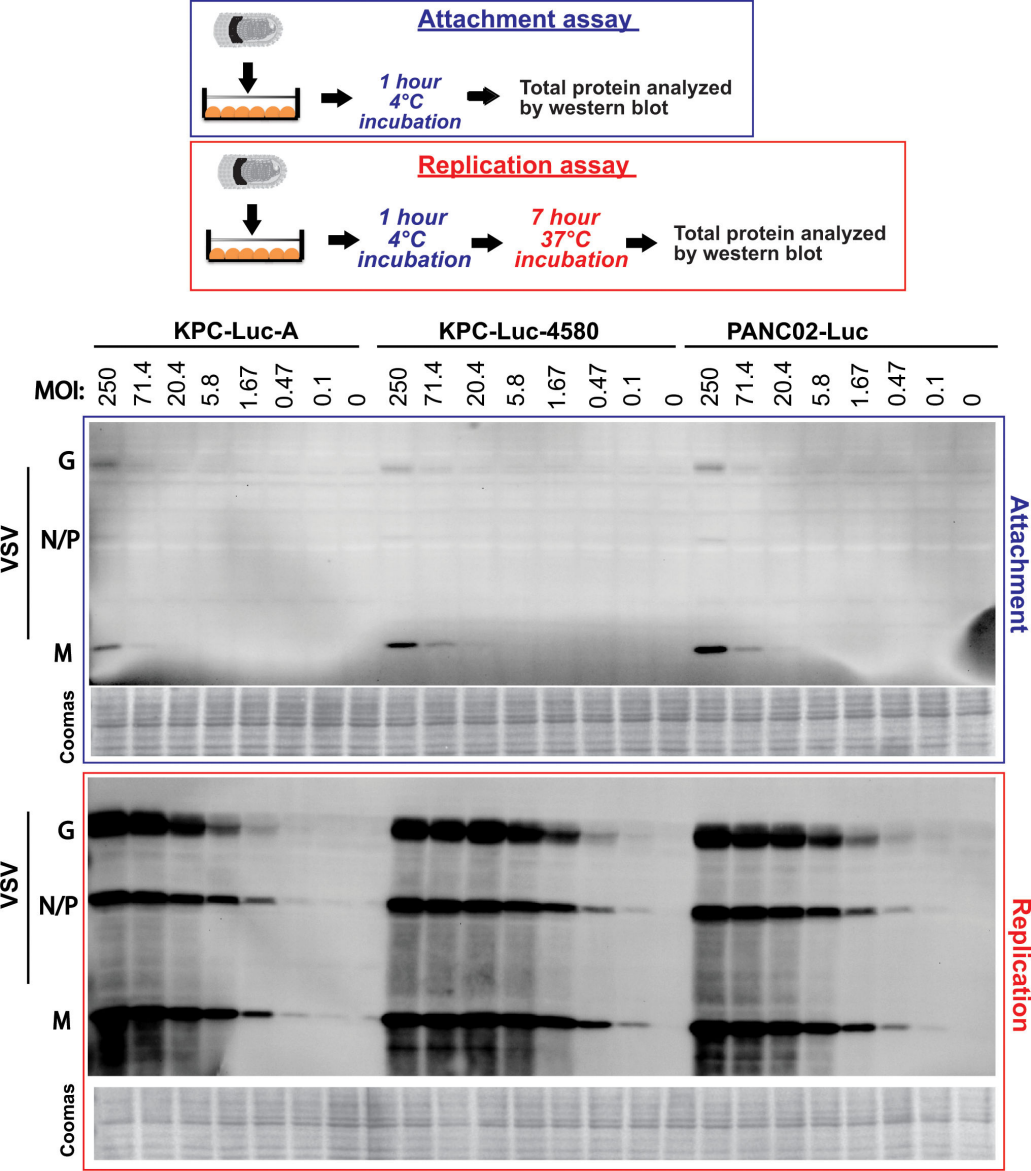
Comparison of VSV-ΔM51-GFP attachment and replication in KPC-Luc-A, KPC-Luc-4580, and PANC02-Luc cells. Cell monolayers were incubated with either virus for 1 h at 4°C (Attachment) or for an additional 7 h at 37°C (Replication). Protein was isolated and analyzed by Western blotting. The MOI is based on virus titration on BHK-21 cells and is indicated at the top. Coomassie blue stain was used to indicate equal loading of samples.

We previously showed that, while abnormal or residual type I IFN antiviral activities play a major role in resistance of some human PDAC cell lines to VSV-ΔM51-GFP, some human PDAC cell lines also show inefficient attachment of VSV (47). Our data in Fig. 6A showed that VSV-ΔM51-GFP or VSV-Mwt-GFP had similar abilities to initiate infection in mouse PDAC cell lines, with the lowest titer in PANC02-Luc and an intermediate titer in KPC-Luc-A. While these differences could be explained by different antiviral status of mouse PDAC cell lines, it could also be due to an inefficient attachment contributing to higher resistance of these cell lines (PANC02-Luc in particular) to VSV-ΔM51-GFP. To examine virus attachment, mouse PDAC cell lines were incubated at various MOIs with purified VSV-ΔM51-GFP at 4°C for 1 h, and the cells were extensively washed to remove any unbound virus and analyzed by Western blotting for virus proteins bound to cells (attachment assay in Fig. 7). At 4°C, the viral particles can only attach to the outside of cells and not enter them. A duplicate set of cells was treated the same way (incubated with the virus at 4°C for 1 h and then extensively washed), but then incubated for more 7 h at 37°C before protein was isolated to examine virus replication (replication assay in Fig. 7). In general, we observed very minor differences in the attachment of VSV-ΔM51-GFP to mouse PDAC cell lines (Fig. 7). Moreover, the highest attachment level was observed in PANC02-Luc cells, which are the most resistant to VSV-ΔM51-GFP. We also noticed very small differences in replication of VSV-ΔM51-GFP in all three mouse cell lines at 8 h p.i. (compare to Fig. 3 for VSV levels at 24 h p.i.), suggesting that VSV-ΔM51-GFP replication is not restricted at earlier stages in mouse PDAC cell lines. These data suggest that the differences in VSV attachment play a minor role in the observed differences in permissiveness of mouse PDAC cell lines to VSV-ΔM51-GFP and also support the hypothesis that the different levels of antiviral signaling play a major role in the resistance of some mouse PDAC cells to VSV-ΔM51-GFP.

### No correlation between resistance of mouse pancreatic cancer cell lines to oncolytic vesicular stomatitis virus and chemotherapy

**Fig 8:**
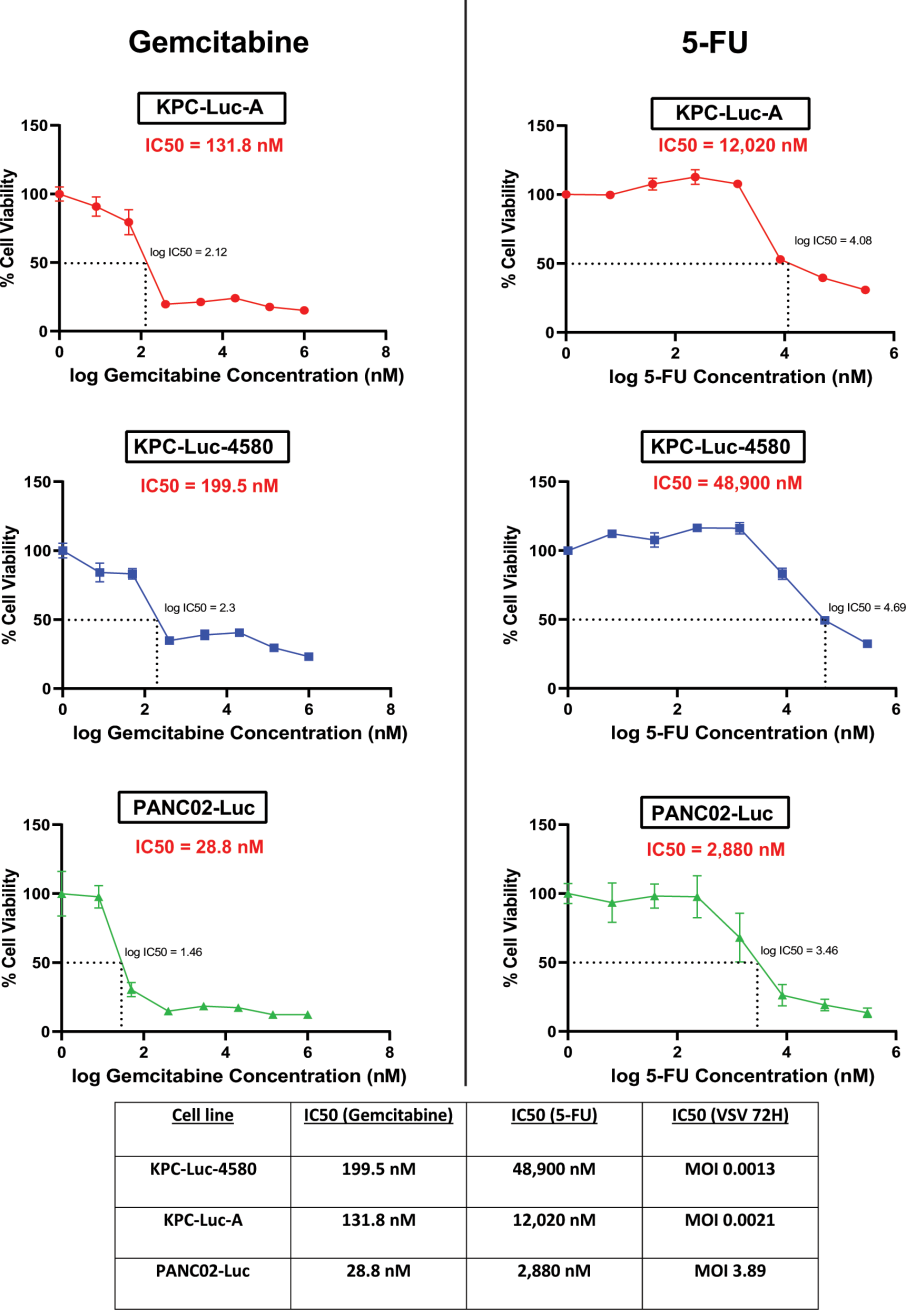
Chemoresistance of mouse PDAC cell lines to gemcitabine and 5-FU. Cells were treated with serial dilutions of either gemcitabine or 5-FU, followed by a cell viability assay 72 h after treatment. IC50 values were calculated using GraphPad prism 9.3.1. Results shown are representative of two independent experiments. The data points and error bars shown represent the means and SEM of the means, respectively (some error bars are too small to be seen in the figures).

We have recently shown that some human PDAC cell lines, which acquire resistance to chemotherapeutical drugs, can also simultaneously develop resistance to OV therapy (48). In addition, although the analyses of 10 different human PDAC cell lines showed no statistically significant correlation between their resistance to gemcitabine and VSV, 4 PDAC cell lines most resistant to VSV were also among 5 PDAC cell lines most resistant to gemcitabine (48). The chemoresistance of PDACs is one of the major reasons for the poor survival outcomes of PDAC patients. We, therefore, sought to investigate the inherent resistance of our mouse PDAC cells against commonly used chemotherapeutic drugs, gemcitabine (2′-deoxy-2′,2′-difliorocytidine monohydrochloride; dFdC; trade name Gemzar) and 5-FU (fluorouracil; trade name Adrucil). Gemcitabine is a deoxycytidine analog that is commonly used in chemotherapeutic regimens for PDAC patients (49). Fluorouracil acts principally as a thymidylate synthase (TS) inhibitor and is used as a chemotherapeutic for a variety of cancers, including PDAC (50). To examine if there is a correlation between the levels of resistance to VSV resistance to both gemcitabine and/or 5-FU, cells were treated with serial dilutions of either gemcitabine or 5-FU, followed by measuring cell viability 72 h later (Fig. 8) and calculation of the IC_50_ for each drug on each cell line that was used as a measure of drug resistance. We found no positive correlation between the level of resistance to VSV and resistance to either gemcitabine or 5-FU. In sharp contrast, PANC02-Luc cells, which are the most resistant mouse PDAC cell line among our three to VSV, demonstrated the lowest IC_50_ for both gemcitabine and 5-FU, at 28.8 and 2,880 nM, respectively. The IC_50_ values for gemcitabine and 5-FU in KPC-Luc-A cells were 131.8 and 12,020 nM. Interestingly, the mouse PDAC cell line most permissive to VSV among our three, KPC-Luc-4580, demonstrated the greatest resistance to gemcitabine and 5-FU with IC50 values of 199.5 and 48,900 nM. These data reveal that the level of chemoresistance in mouse PDAC cell lines does not correlate with the efficacy of VSV-based OV-therapy and, promisingly, suggest that chemoresistant tumors can be good targets for OV therapy.

### Karyotype and exome analyses of mouse pancreatic cancer cell lines

**Fig 9:**
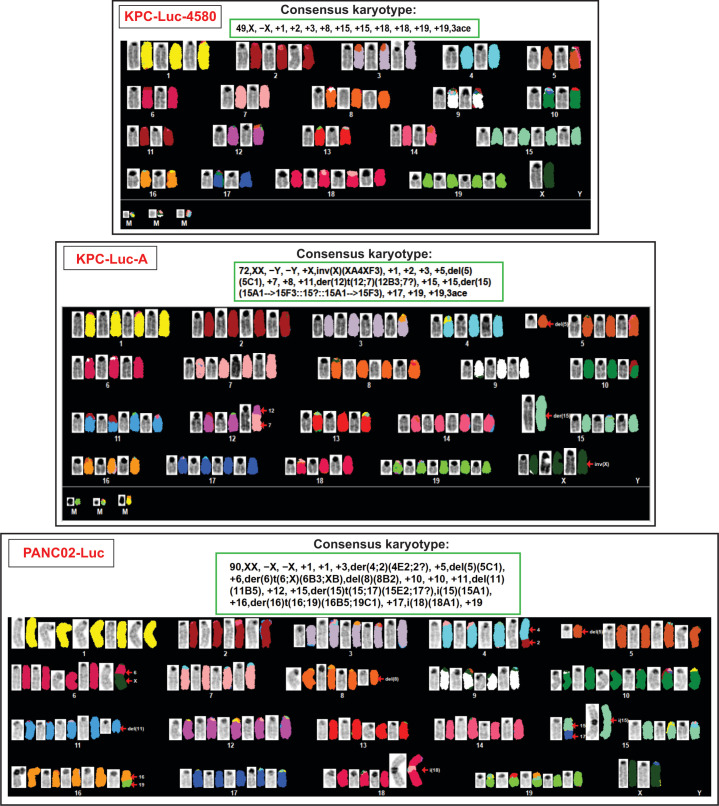
G-band and spectral karyotyping (SKY) of mouse PDAC cell lines. Adherent cells were harvested with colcemid arrest, treatment with 0.75 M KCl hypotonic solution, and fixation with 3:1 methanol:acetic acid. The resulting cells were spread onto glass slides according to standard cytogenetic protocols. A spectral karyotyping (SKY) slide was processed according to the manufacturer’s protocol [Applied Spectral Imaging (ASI)]. SKY uses a unique combination of five fluorescent dyes to paint all 24 chromosomes. Seven metaphase cells per sample were examined by SKY using the Olympus BX61 microscope with DAPI and SKY fluorescence filter sets. G-band and SKY metaphase cells were imaged and karyotyped using ASI software.

Although all three tested mouse PDAC cell lines are widely used as models to study PDAC biology and treatments for PDAC *in vivo*, to our knowledge, they have never been tested in detail for overall chromosomal abnormalities or all genomic mutations in protein-coding sequences. To examine the karyotype of these cells, each cell line was grown to 70% confluence and sent to the Cytogenomics Core Laboratory at the University of Minnesota for SKY. We found that each cell line contained numerous chromosomal aberrations (Fig. 9, the “consensus karyotype” is shown in the green box; a complete analysis is shown in Fig. S1). KPC-Luc-4580, KPC-Luc-A, and PANC02-Luc cells exhibited overall chromosome count ranges of 48–51, 65–72, and 97–90, respectively. Interestingly, PANC02-Luc and then KPC-Luc-A cell lines displayed the greatest degree of chromosomal aberration, with karyotypes more closely resembling hypertetraploid and hypertriploid genomes, respectively. Of note, for each cell line, the level of chromosomal aberration seems to correlate with the level of resistance to VSV. However, while this potential correlation is interesting, we do not have any data suggesting that it is causative, and investigating specific chromosomal aberrations and how they might affect resistance to VSV is beyond the scope of this study. Rather, these data are important for future studies in understanding major genetic differences between model mouse PDAC cell lines.

**Fig 10:**
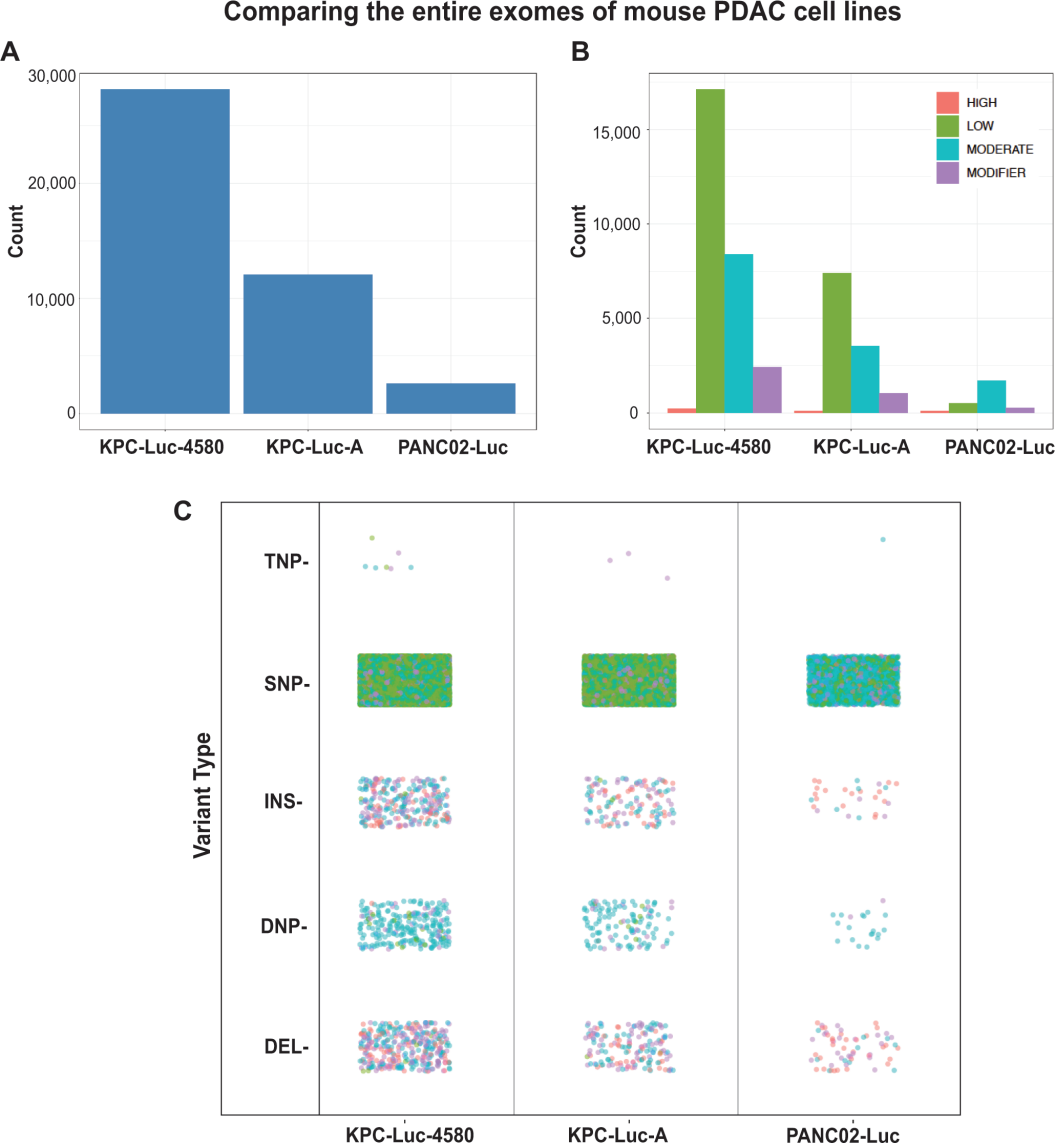
Exome analysis of all genes in three mouse PDAC cell lines. Abbreviations are as follows: SNP (single-nucleotide polymorphisms), DNP (double nucleotide polymorphism), TNP (triple nucleotide polymorphism), INS (insertion), DEL (deletion). Mutations were annotated according to AZENTA Life Sciences Sequence Ontology pipeline, which leverages the Ensembl database of calculated variant consequences. The Impact rating uses the same qualifiers as SnpEff and SnpSift software (https://pcingola.github.io/SnpEff/se_introduction/) and can be interpreted as the following: High (disruptive, truncating, loss of function of protein), Low (harmless or unchanging to protein), Moderate (non-disruptive, but may have an effect), and Modifier (changes in non-coding regions or in regions where function and impact is unable to be assessed).

We have shown in this study so far that each of the three mouse PDAC cell lines is different in their permissiveness to VSV and their antiviral expression profiles. Therefore, we hypothesized that more VSV-permissive and more IFN-deficient cell lines will harbor more mutations in genes associated with innate immunity/antiviral response. To examine genomic mutations in protein-coding sequences of these three mouse PDAC cell lines, cell pellets were sent to Azenta US for DNA extraction, exome sequencing, and exome analysis (Fig. 10; Table S1; the entire exome data were deposited to the GenBank SRA database under BioProject PRJNA944630). Interestingly, we found that both KPC cell lines exhibited a far greater number of mutations compared to PANC02-Luc cells (Fig. 10A). The mutations were further classified based on genome impact (Fig. 10B) as “high” (mutations affecting splice sites, start and stop codons), “moderate” (non-synonymous variations), “low” (synonymous variations), and “modifier” (variations in non-coding regions, e.g., upstream, downstream, intergenic, and UTR regions). In both KPC cell lines, the majority of mutations are classified as low impact, followed by moderate, modifier, and high, respectively. In PANC02-Luc cells, the majority of mutations were classified as moderate impact, followed by low, modifier, and high, respectively. The mutations were then further broken down by variant type (Fig. 10C). In all cell lines, the majority of mutations were single-nucleotide polymorphisms (SNPs). Consistent with Fig. 10B, the majority of SNPs in PANC02-Luc cells appear to be of moderate impact, in contrast to both KPC cell lines where the majority of SNPs are low impact. After SNPs, the greatest number of mutations in each cell line were deletions (DEL), followed by insertions (INS), double nucleotide polymorphisms (DNP), and triple nucleotide polymorphisms (TNP), respectively. Most of the high-impact mutations in each cell line were INSs and DELs.

**Fig 11:**
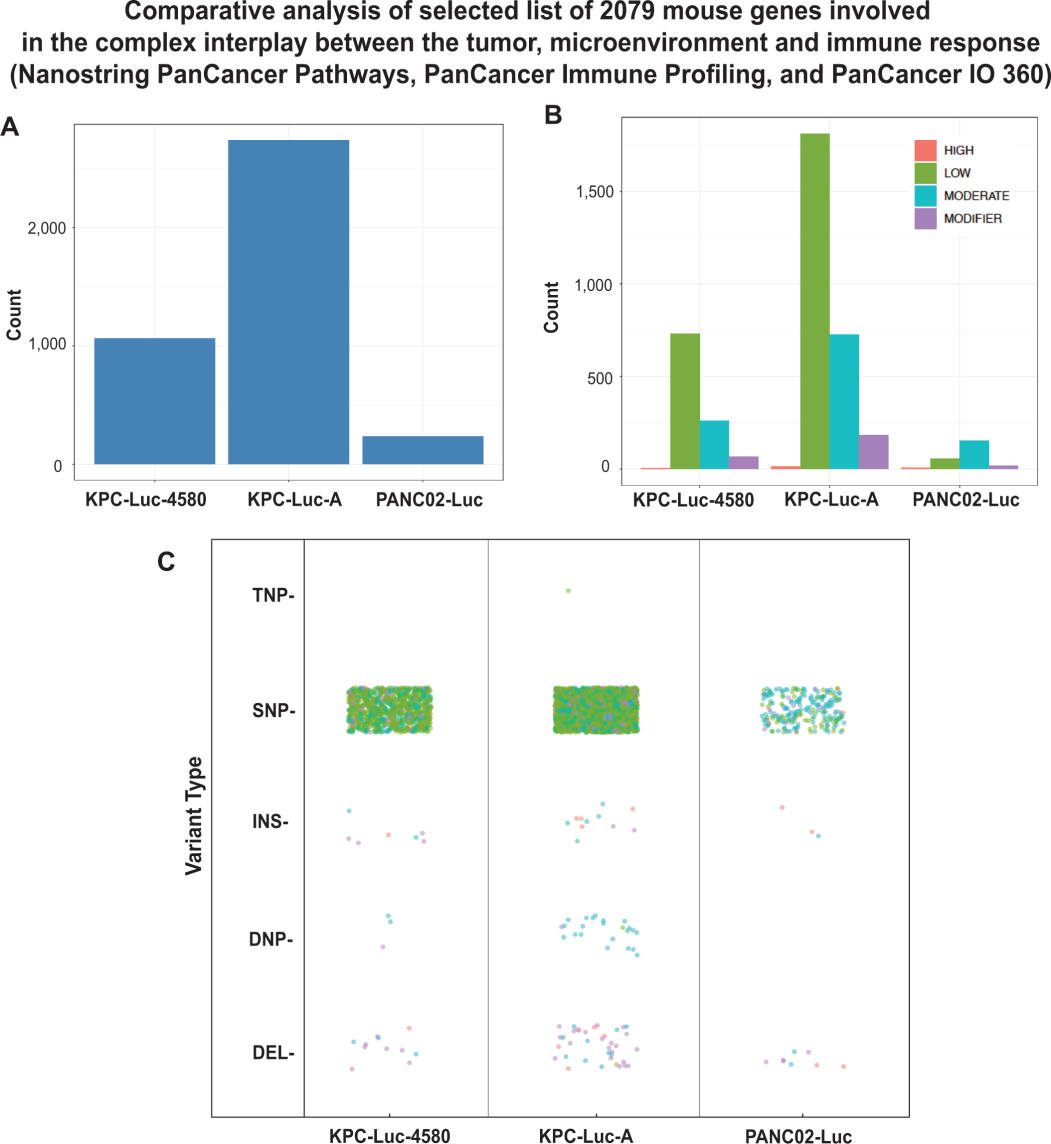
Exome analysis of 2,079 genes involved in tumor, microenvironment and immune response variant annotations based on Azenta Genewiz pipeline based on Ensembl Sequence Ontology. SNP (single-nucleotide polymorphisms), DNP (double nucleotide polymorphism), TNP (triple nucleotide polymorphism), INS (insertion), DEL (deletion). Mutations were annotated according to AZENTA Life Sciences Sequence Ontology pipeline, which leverages the Ensembl database of calculated variant consequences. The Impact rating uses the same qualifiers as SnpEff and SnpSift software (https://pcingola.github.io/SnpEff/se_introduction/) and can be interpreted as the following: High (disruptive, truncating, loss of function of protein), Low (harmless or unchanging to protein), Moderate (non-disruptive, but may have an effect), and Modifier (changes in non-coding regions or in regions where function and impact is unable to be assessed).

To focus on the genes associated with cancer and innate immunity, we utilized the NanoString PanCancer IO 360 Panel, PanCancer Pathways Panel, and the PanCancer Immune Profiling Panel to generate a list of cancer and immune genes of interest. Overall, we found over 2,000 mutations in genes associated with cancer and innate immunity (Fig. 11; Table S2). Interestingly, we found that both KPC cell lines exhibited a dramatically greater number of mutations (KPC-Luc-4580: 12,065, KPC-Luc-A: 28,131) compared to PANC02-Luc cells (2,609) (Fig. 11A). In both KPC cell lines, the majority of mutations are classified as low impact, followed by moderate, modifier, and high, respectively. In PANC02-Luc cells, the majority of mutations were classified as moderate impact, followed by low, modifier, and high, respectively (Fig. 11B). Figure 11C shows that the majority of mutations were SNPs, with the least mutation type being TNPs. Importantly, we found that both KPC cell lines contain the classical PDAC Trp53R172H and KrasG12D driver mutations. As previously reported, we found no mutations in Trp53 and Kras in PANC02-Luc cells (51). However, we found that only PANC02-Luc cells contain a nonsense mutation in another important PDAC gene, Smad4 (Smad4 mutation absent in both KPC cell lines). Consistent with our hypothesis, in both KPC cell lines, we found mutations in many key antiviral genes (Table 1). Notably, we found multiple identical missense mutations in the tyrosine kinase 2 (TYK2) gene, a critical upstream modulator of type I IFN signaling. We also found in both KPC cell lines identical missense mutations in the JAK2 and JAK3 genes, which are critical upstream modulators of innate and adaptive immunity, respectively. Such identical mutations suggest that these mutations are important for the overall fitness of these cells. Additionally, these mutations in innate immunity-associated genes may be responsible in part for the higher permissiveness of KPC cells to VSV infection compared to PANC02-Luc (Table 1; also see Table S2 for other mutations). While specific analysis of the role of each mutation in these cell lines is beyond the scope of this study, the exome sequencing data provide valuable information for future studies using these mouse PDAC cell lines.

**TABLE 1:** Selected exome missense mutations in type I IFN pathway genes

Gene	Cell line	Mutation
TYK2	KPC-Luc-4580	p.F516L
TYK2	KPC-Luc-4580	p.K382E
TYK2	KPC-Luc-4580	p.H261R
TYK2	KPC-Luc-A	p.F516L
TYK2	KPC-Luc-A	p.K382E
TYK2	KPC-Luc-A	p.H261R
STAT4	PANC02-Luc	p.S72F
OAS2	KPC-Luc-A	p.C118W
OAS2	PANC02-Luc	p.Q232R
OAS3	KPC-Luc-A	p.H897Q
OAS3	PANC02-Luc	p.L993R
IRF2	KPC-Luc-A	p.D295E
IFI203	KPC-Luc-A	p.R198Q
IFI203	KPC-Luc-A	p.I52M
IFI35	KPC-Luc-A	p.R108W
IFIH1	KPC-Luc-A	p.K647E
IFIH1	KPC-Luc-A	p.Q434H
IFIT1	PANC02-Luc	p.P51A
IFIT2	PANC02-Luc	p.R383H
TRIM21	KPC-Luc-A	p.E318K
USP18	KPC-Luc-A	p.V115L
ISG20	PANC02-Luc	p.A9P
JAK2	KPC-Luc-A	p.K575E
JAK2	KPC-Luc-4580	p.K575E
JAK3	KPC-Luc-A	p.G699C
JAK3	KPC-Luc-A	p.S1032R
JAK3	KPC-Luc-4580	p.G699C
JAK3	KPC-Luc-4580	p.S1032R
TLR9	KPC-Luc-A	p.T325N
TLR9	KPC-Luc-A	p.L378S
TLR9	KPC-Luc-A	p.T573A
TLR9	KPC-Luc-A	p.Q579H
TLR2	KPC-Luc-4580	p.M82I
TLR4	PANC02-Luc	p.Q42E
TLR4	PANC02-Luc	p.D462N
TLR5	KPC-Luc-A	p.R86Q
TLR5	KPC-Luc-A	p.G857A
TLR8	KPC-Luc-A	p.R578S

## DISCUSSION

Preclinical models of PDAC are vital for understanding the biology of PDAC and are platforms for developing novel strategies against PDAC, one of the deadliest forms of cancer, which has been the number 4 cause of cancer-related deaths in the USA since the 1970s (1, 2). The ideal PDAC model system should address several key features of PDAC, including intertumoral heterogeneity affecting the efficacy of OV-based therapy against different PDACs, and the ability to test new therapies in immunocompetent mice.

In this study, we examined phenotypically and genotypically three commonly used, allograftable mouse PDAC cell lines: a widely used PANC02-Luc (derived from chemically induced PDAC), and two KPC cell lines originated from PDACs developed in different modified KPC mice encoding major driver mutations KrasG12D and Trp53R172H in the pancreas. We found that the three mouse PDAC cell lines were genotypically and phenotypically distinct and showed very different permissiveness to OV-based therapy and, therefore, can serve as promising cell lines to use and address intertumoral heterogeneity *in vivo* in immunocompetent mice.

We found that each of the three mouse PDAC cell lines exhibited varying levels of permissiveness to VSV infection and VSV-mediated cell killing (Fig. 1 and 2). We also showed that the level of cellular permissiveness to VSV infection and VSV-mediated cell killing correlated with levels of type I IFN antiviral signaling (Fig. 3 to 5), including their abilities to activate STAT1 in response to VSV infection (Fig. 3). Another piece of evidence demonstrating the differential levels of type I IFN antiviral signaling between the tested cell lines is highlighted by their responsiveness to added IFNα (Fig. 4). The mIFNα IC_50_ values for each cell line represent the relative ability of each cell line to respond to IFN. Between the three tested mouse cell lines, PANC02-Luc displayed the lowest mIFNα IC_50_ (33.1 U/mL), versus the highest IC_50_ in KPC-Luc-4580 cells (354.8 U/mL), and intermediate IC_50_ of 61.6 U/mL for KPC-Luc-A. This means that it takes almost 11 times the amount of mIFNα to bring KPC-Luc-4580 cells to the same level of type I IFN antiviral signaling as PANC02-Luc cells. We further highlight the differential levels of type I (and III) IFN antiviral signaling between these cell lines in Fig. 5, where we show the dramatic differences in secreted IFNβ and IFNγ after VSV infection. We also observed a dramatic increase in the size of plaques, better replication kinetics and oncolysis for non-attenuated and less sensitive to type I IFN signaling VSV-Mwt-GFP (compared to VSV-ΔM51-GFP) in KPC-Luc-A and especially in PANC02-Luc (Fig. 6). Also, our data show no significant differences between mouse PDAC cell lines in VSV-ΔM51-GFP attachment or virus replication at 8 h p.i., suggesting that VSV-ΔM51-GFP replication is not restricted at earlier stages in mouse PDAC cell lines. Taken together, our data support the hypothesis that the ability to mount functional antiviral signaling is a major determinant of permissiveness of mouse PDAC cell lines to VSV-ΔM51-GFP.

Of the three tested mouse cell lines, PANC02-Luc exhibited the highest level of resistance to VSV. To speculate potential reasons why this cell line displays the highest levels of type I IFN antiviral signaling, we first looked at how the cell line was generated. PANC02-Luc is a PDAC cell line isolated from a tumor generated chemically using a potent mutagen, 3-MC (29). 3-MC is highly carcinogenic and has been used to induce cancer in rodents since the mid-1900s (52). The use of 3-MC likely led to DNA damage and mutations in the pancreas (53). Moreover, a previous study showed that the transformation of immortalized human uroepithelial cells by 3-MC increases IFN-stimulated genes (ISGs) expression, including expression of well-known antiviral genes, such as 2′−5′ OAS and MxA (54). In general, there is a growing body of studies (55 – 57), including previous studies from our lab (58), suggesting that cancer cells may upregulate antiviral signaling pathways to confer protection against radiation therapy and chemotherapy. The high degree of DNA damage caused by 3-MC in the pancreas may have led to an upregulation of antiviral signaling in PANC02-Luc cells. Consistent with this idea, we show evidence of higher DNA damage in PANC02-Luc cells in Fig. 9, where the karyotype analysis reveals a highly aberrated, hyper-tetraploid genome. Additionally, we showed that PANC02-Luc cells display the slowest doubling time among the mouse PDAC cell lines tested in this study, which correlated with their increased level of resistance to VSV. As well, the doubling time for KPC-Luc A and KPC-Luc-4580 cells also corresponded to their levels of resistance to VSV-ΔM51-GFP. It is understood that cancer cells typically downregulate antiviral signaling as these pathways are antiproliferative and pro-apoptotic (13). Also, in our previous studies, we demonstrated that cells in the G_2_/M cell cycle phase are transcriptionally repressed, leading to a decreased ability of cells to mount antiviral responses (33). Therefore, we speculate that cells progressing through the cell cycle more quickly may allow for better VSV-ΔM51-GFP replication.

Interestingly, while PANC02-Luc cells showed the greatest degree of chromosomal aberration, they exhibited the smallest number of whole exome mutations (Fig. 10), as well as mutations in 2,079 mouse genes known to be involved in the complex interplay between the tumor, microenvironment, and immune response (Nanostring PanCancer Pathways, PanCancer Immune Profiling, and PanCancer IO 360) (Fig. 11; Table S2). KPC-Luc-A contained the highest number of mutations, respectively, with both KPC cell lines harboring mutations in multiple important antiviral-associated genes, such as TYK2 and JAK2 (Table 1). These mutations in genes that encode important antiviral signaling proteins may contribute to the higher permissiveness of KPC cells to VSV compared to PANC02-Luc cells.

Especially significant could be the lack of tumor-associated KRAS mutations in PANC02-Luc cells. Unlike PANC02-Luc cells, KPC-Luc-A and KPC-Luc-4580 cells were isolated from tumors that were formed spontaneously in GEMMs with the driver mutations KrasG12D and Trp53R172H. Higher permissiveness of KPC-Luc-A and KPC-Luc-4580 (compared to PANC02-Luc) to VSV could be also explained by the presence of KrasG12D driver mutation in these 2 KPC cell lines, while it is absent in PANC02-Luc (51). Several recent studies demonstrated that tumor-associated KRAS mutations, that result in abnormal activation of the RAS/Raf1/MEK/ERK pathway, can lead to multiple defects in the type I IFN signaling, thus making KRAS^MUT^ cancer cells more permissive to VSV and other OVs (59 – 63). For example, Ras/MEK suppresses the basal expression levels of key components of the type I IFN signaling pathway, hence leading to cellular impairment of IFN-induced antiviral responses (64). Additionally, Ras/MEK regulates the activity of positive (IRF1 and Sp3) and negative (NF-κB) regulators of ISGF3 (61). Therefore, IFN-inducible genes that require up or downregulation of a co-regulator for their expression could be suppressed in cells with activated Ras/MEK. While our future studies will address this possibility experimentally, it is important to note that 90–95% of pancreatic cancers have a KRAS mutation (65), which makes them intrinsically more permissive to VSV and other OVs via KRAS^MUT^-mediated inhibition of antiviral signaling.

Interestingly, we found that KPC-Luc-A and KPC-Luc-4580 cell lines, even though isolated from PDAC tumors generated from cells that had the same driver mutations, exhibited differential responses to VSV infection. Multiple possible reasons for the differences in phenotypes and genotypes between KPC-Lu-A and KPC-Luc-4580 include: (i) different environmental factors and selective pressures during tumor development in mice; (ii) different selective pressures throughout cell culture adaptation, and (iii) random genetic changes. To our knowledge, the only difference in the original engineering design between KPC-Luc-A and KPC-Luc-4580 cell lines was the method by which each cell line was engineered to express luciferase (Fig. 1A). In KPC-Luc-A mice, after PDAC cells were isolated from the tumor, the luciferase gene was incorporated into the genome via lentivirus transduction. However, the generation of KPC-Luc-4580 mice involved an additional cross with a ROSA26luc/+ mouse, leading to luciferase expression from the ROSA26 locus. A possible explanation for the differences between KPC cell lines could be that although parental mice had the same PDAC driver mutations, subsequent alternative mutations likely developed. It is most likely that the reason for genetic differences between the KPC cell lines is a combination of each of the factors mentioned above. Evidence for genomic differences between KPC cell lines is dramatically presented in Fig. 9, where we show multiple different chromosomal aberrations between the KPC cell lines. Genomic differences are also highlighted at the exome level, where both KPC cell lines contain a far greater number of mutations in antiviral genes compared to PANC02-Luc cells (Fig. 11).

Whatever the reason for the phenotypic and genotypic differences between the tested KPC cell lines, these differences help contribute to their clinical translatability, as they likely better represent characteristic heterogeneity among and within PDAC patients.

As VSV-based OV therapy becomes a more commonly tested therapeutic option in clinical trials (Clinicaltrials.gov trials NCT01628640, NCT03120624, NCT04046445, NCT03865212, NCT03017820, and NCT03647163), it is necessary to better understand how the effectiveness of VSV-based OV therapy is affected by the chemoresistance status of cancer cells. It has been observed in our previous studies and others (48, 55, 57, 66) that the level of chemotherapeutic drug resistance may correlate with the level of antiviral signaling in cancer cells. This phenomenon is important to consider when determining the best therapeutic regimen for cancer patients. In this study, we hypothesized that the level of resistance to two commonly used chemotherapeutic drugs for PDAC, gemcitabine, and 5-FU, would positively correlate with the level of resistance to VSV. Surprisingly, we found that there was an inverse correlation between VSV resistance and drug resistance (Fig. 8), where the most resistant cell line to VSV (PANC02-Luc) had the lowest IC_50_ for both gemcitabine and 5-FU, and the least resistant cell line to VSV (KPC-Luc-4580) had the highest IC_50_ for gemcitabine and 5-FU. These data suggest that even if PDAC in patients is inherently chemoresistant, VSV-based OV therapy remains a viable treatment option.

**Fig 12:**
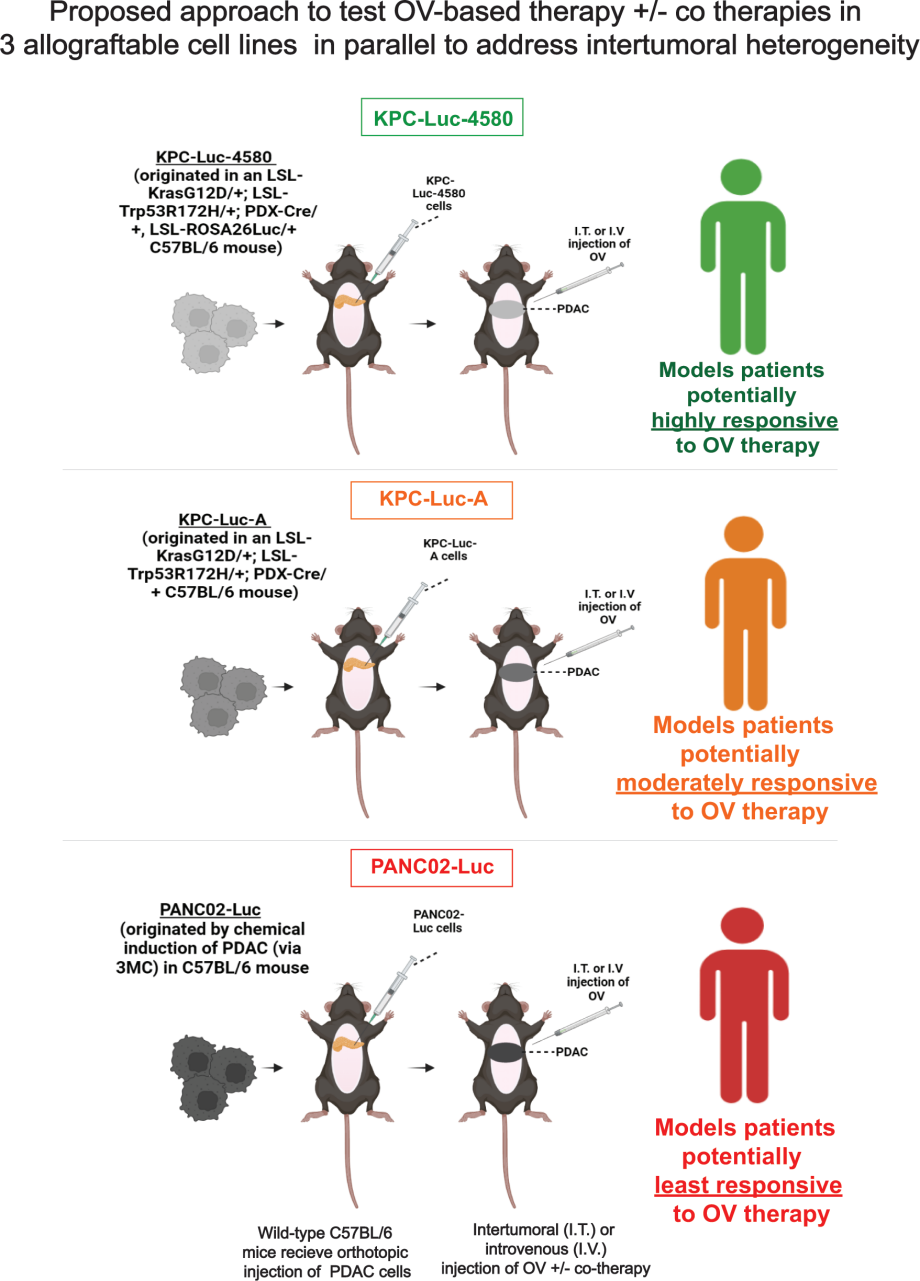
Proposed novel platform to study OV-based therapies against phenotypically different PDACs in immunocompetent mice. This model illustrates our proposed approach to test OV-based therapy ± co-therapies in three allograftable cell lines in parallel to address intertumoral heterogeneity.

The ideal PDAC mouse model system should address both the intertumoral heterogeneity and the ability to detect and evaluate innate and adaptive immune responses against both tumor and OV. In Fig. 12, we outline one such model based on our discoveries. We propose that multiple different mouse PDAC cell lines should be tested in parallel, ideally with each exhibiting varying levels of response to OV therapy to better recapitulate tumor heterogeneity. KPC cell lines (as outlined in this study) are great candidates as they express luciferase (for tumor tracking), are C57BL/6 background (allowing for studies in wild-type immunocompetent mice), originated spontaneously in KPC mice via the same driver mutations as in human disease, and show different responses to OV-therapy and chemotherapeutics. While KPC cell lines contain the same driver mutations observed in ~85% of all PDAC (67), PANC02-Luc cells, which lack these mutations, are clinically relevant as they represent the small percent of PDACs without the classical Kras and P53 driver mutations. Figure 12 illustrates how we envision these cell lines could be used to address intertumoral heterogeneity to OVs ± co-therapies. Each cell line represents either the most permissive to OV, moderately permissive to OV, or least permissive to OV. Importantly, novel OVs and co-therapies should be tested in parallel. The success of newly tested OVs and other co-therapies can be evaluated via a scoring system based on whether the therapy was successful in 0/3, 1/3, 2/3, or 3/3 tumor types. As illustrated in Fig. 12, wild-type C57BL/6 mice should be used to study the role of the adaptive immune response. Additionally, although a challenging approach, tumor cells should be injected orthotopically into the pancreas to allow investigations into the TME and metastasis.

In conclusion, our study provides essential data about three allograftable model mouse PDAC cell lines and proposes a novel platform to study OV-based therapies against phenotypically and genotypically distinct PDACs in immunocompetent mice. This study will be useful for ongoing and future studies in the field of PDAC therapeutics. Although this study focused on VSV as the oncolytic agent, alternative OVs can be tested using the model cell lines characterized in this study.

## Data Availability

The exome data were deposited to the GenBank Sequence Read Archive (SRA) database under BioProject PRJNA944630. Exome analysis of three murine pancreatic cancer cell lines under the following BioSample and SRA accessions: PANC02-Luc, KPC-Luc-A, and KPC-Luc-4580.

## SUPPLEMENTAL MATERIAL

Supplemental Figure S1 jvi.01005-23-s0001.pdf Detailed karyotype analysis of mouse PDAC cell lines.

Supplemental Table S1 jvi.01005-23-s0002.xlsx Cytokine array data.

Supplemental Table S2 jvi.01005-23-s0003.xlsx Exome analysis identifies mutations in genes associated with cancer and innate immunity.

ASM does not own the copyrights to Supplemental Material that may be linked to, or accessed through, an article. The authors have granted ASM a non-exclusive, world-wide license to publish the Supplemental Material files. Please contact the corresponding author directly for reuse.

## References

[1] Siegel RL , Miller KD , Wagle NS , Jemal A . 2023. Cancer statistics, 2023. CA Cancer J Clin 73:17–48. doi:10.3322/caac.21763 36633525

[2] Mizrahi JD , Surana R , Valle JW , Shroff RT . 2020. Pancreatic cancer. Lancet 395:2008–2020. doi:10.1016/S0140-6736(20)30974-0 32593337

[3] Shalhout SZ , Miller DM , Emerick KS , Kaufman HL . 2023. Therapy with oncolytic viruses: progress and challenges. Nat Rev Clin Oncol 20:160–177. doi:10.1038/s41571-022-00719-w 36631681

[4] Engeland CE , Bell JC . 2020. Introduction to oncolytic virotherapy. Methods Mol Biol 2058:1–6. doi:10.1007/978-1-4939-9794-7_1 31486028

[5] Felt SA , Grdzelishvili VZ . 2017. Recent advances in vesicular stomatitis virus-based oncolytic virotherapy: a 5-year update. J Gen Virol 98:2895–2911. doi:10.1099/jgv.0.000980 29143726 PMC5845697

[6] Liu G , Cao W , Salawudeen A , Zhu W , Emeterio K , Safronetz D , Banadyga L . 2021. Vesicular stomatitis virus: from agricultural pathogen to vaccine vector. Pathogens 10:1092. doi:10.3390/pathogens10091092 34578125 PMC8470541

[7] Hastie E , Grdzelishvili VZ . 2012. Vesicular stomatitis virus as a flexible platform for oncolytic virotherapy against cancer. J Gen Virol 93:2529–2545. doi:10.1099/vir.0.046672-0 23052398 PMC4091291

[8] Hastie E , Cataldi M , Marriott I , Grdzelishvili VZ . 2012. Understanding and altering cell tropism of vesicular stomatitis virus. Virus Res 176:16–32. doi:10.1016/j.virusres.2013.06.003 PMC3865924 23796410

[9] Finkelshtein D , Werman A , Novick D , Barak S , Rubinstein M . 2013. LDL receptor and its family members serve as the cellular receptors for vesicular stomatitis virus. Proc Natl Acad Sci U S A 110:7306–7311. doi:10.1073/pnas.1214441110 23589850 PMC3645523

[10] Stojdl DF , Lichty B , Knowles S , Marius R , Atkins H , Sonenberg N , Bell JC . 2000. Exploiting tumor-specific defects in the interferon pathway with a previously unknown Oncolytic virus. Nat Med 6:821–825. doi:10.1038/77558 10888934

[11] Lichty BD , Power AT , Stojdl DF , Bell JC . 2004. Vesicular stomatitis virus: re-inventing the bullet. Trends Mol Med 10:210–216. doi:10.1016/j.molmed.2004.03.003 15121047

[12] Zhang K , Matsui Y , Hadaschik BA , Lee C , Jia W , Bell JC , Fazli L , So AI , Rennie PS . 2010. Down-regulation of type I interferon receptor sensitizes bladder cancer cells to vesicular stomatitis virus-induced cell death. Int J Cancer 127:830–838. doi:10.1002/ijc.25088 19957332

[13] Shi W , Yao X , Fu Y , Wang Y . 2022. Interferon-α and its effects on cancer cell apoptosis. Oncol Lett 24:235. doi:10.3892/ol.2022.13355 35720476 PMC9185151

[14] Holbrook MC , Goad DW , Grdzelishvili VZ . 2021. Expanding the spectrum of pancreatic cancers responsive to vesicular stomatitis virus-based oncolytic virotherapy: challenges and solutions. Cancers (Basel) 13:1171. doi:10.3390/cancers13051171 33803211 PMC7963195

[15] He M , Henderson M , Muth S , Murphy A , Zheng L . 2020. Preclinical mouse models for immunotherapeutic and non-immunotherapeutic drug development for pancreatic ductal adenocarcinoma. Ann Pancreat Cancer 3:7. doi:10.21037/apc.2020.03.03 32832900 PMC7440242

[16] Kong KW , Guo M , Liu YF , Zheng JM . 2020. Progress in animal models of pancreatic ductal adenocarcinoma. J Cancer 11:1555–1567. doi:10.7150/jca.37529 32047562 PMC6995380

[17] Liu X , Wang W , Liu X , Zhang Z , Yu L , Li R , Guo D , Cai W , Quan X , Wu H , Dai M , Liang Z . 2022. Multi-omics analysis of intra-tumoural and inter-tumoural heterogeneity in pancreatic ductal adenocarcinoma. Clin Transl Med 12:e670. doi:10.1002/ctm2.670 35061935 PMC8782496

[18] Hastie E , Cataldi M , Moerdyk-Schauwecker MJ , Felt SA , Steuerwald N , Grdzelishvili VZ . 2016. Novel biomarkers of resistance of pancreatic cancer cells to oncolytic vesicular stomatitis virus. Oncotarget 7:61601–61618. doi:10.18632/oncotarget.11202 27533247 PMC5308675

[19] Moerdyk-Schauwecker M , Shah NR , Murphy AM , Hastie E , Mukherjee P , Grdzelishvili VZ . 2013. Resistance of pancreatic cancer cells to oncolytic vesicular stomatitis virus: role of type I interferon signaling. Virology 436:221–234. doi:10.1016/j.virol.2012.11.014 23246628 PMC3544977

[20] Murphy AM , Besmer DM , Moerdyk-Schauwecker M , Moestl N , Ornelles DA , Mukherjee P , Grdzelishvili VZ . 2012. Vesicular stomatitis virus as an oncolytic agent against pancreatic ductal adenocarcinoma. J Virol 86:3073–3087. doi:10.1128/JVI.05640-11 22238308 PMC3302313

[21] Hingorani SR , Wang L , Multani AS , Combs C , Deramaudt TB , Hruban RH , Rustgi AK , Chang S , Tuveson DA . 2005. Trp53R172H and KrasG12D cooperate to promote chromosomal instability and widely metastatic pancreatic ductal adenocarcinoma in mice. Cancer Cell 7:469–483. doi:10.1016/j.ccr.2005.04.023 15894267

[22] Stopczynski RE , Normolle DP , Hartman DJ , Ying H , DeBerry JJ , Bielefeldt K , Rhim AD , DePinho RA , Albers KM , Davis BM . 2014. Neuroplastic changes occur early in the development of pancreatic ductal adenocarcinoma. Cancer Res 74:1718–1727. doi:10.1158/0008-5472.CAN-13-2050 24448244 PMC4036226

[23] Gilabert M , Calvo E , Airoldi A , Hamidi T , Moutardier V , Turrini O , Iovanna J . 2014. Pancreatic cancer-induced cachexia is jak2-dependent in mice. J Cell Physiol 229:1437–1443. doi:10.1002/jcp.24580 24648112

[24] Torres MP , Rachagani S , Souchek JJ , Mallya K , Johansson SL , Batra SK . 2013. Novel pancreatic cancer cell lines derived from genetically engineered mouse models of spontaneous pancreatic adenocarcinoma: applications in diagnosis and therapy. PLoS One 8:e80580. doi:10.1371/journal.pone.0080580 24278292 PMC3835415

[25] Wollmann G , Rogulin V , Simon I , Rose JK , van den Pol AN . 2010. Some attenuated variants of vesicular stomatitis virus show enhanced oncolytic activity against human glioblastoma cells relative to normal brain cells. J Virol 84:1563–1573. doi:10.1128/JVI.02040-09 19906910 PMC2812324

[26] Das SC , Nayak D , Zhou Y , Pattnaik AK . 2006. Visualization of intracellular transport of vesicular stomatitis virus nucleocapsids in living cells. J Virol 80:6368–6377. doi:10.1128/JVI.00211-06 16775325 PMC1488946

[27] Erstad DJ , Sojoodi M , Taylor MS , Ghoshal S , Razavi AA , Graham-O’Regan KA , Bardeesy N , Ferrone CR , Lanuti M , Caravan P , Tanabe KK , Fuchs BC . 2018. Orthotopic and heterotopic murine models of pancreatic cancer and their different responses to FOLFIRINOX chemotherapy. Dis Model Mech 11:dmm034793. doi:10.1242/dmm.034793 29903803 PMC6078400

[28] Manuel ER , Chen J , D’Apuzzo M , Lampa MG , Kaltcheva TI , Thompson CB , Ludwig T , Chung V , Diamond DJ . 2015. Salmonella-based therapy targeting indoleamine 2,3-dioxygenase coupled with enzymatic depletion of tumor hyaluronan induces complete regression of aggressive pancreatic tumors. Cancer Immunol Res 3:1096–1107. doi:10.1158/2326-6066.CIR-14-0214 26134178 PMC4561205

[29] Corbett TH , Roberts BJ , Leopold WR , Peckham JC , Wilkoff LJ , Griswold DP , Schabel FM . 1984. Induction and chemotherapeutic response of two transplantable ductal adenocarcinomas of the pancreas in C57Bl/6 mice. Cancer Res 44:717–726. 6692374

[30] Iwamura T , Katsuki T , Ide K . 1987. Establishment and characterization of a human pancreatic cancer cell line (SUIT-2) producing carcinoembryonic antigen and carbohydrate antigen 19-9. Jpn J Cancer Res 78:54–62. 3102439

[31] Metzgar RS , Gaillard MT , Levine SJ , Tuck FL , Bossen EH , Borowitz MJ . 1982. Antigens of human pancreatic adenocarcinoma cells defined by murine monoclonal antibodies. Cancer Res 42:601–608. 7034925

[32] Yunis AA , Arimura GK , Russin DJ . 1977. Human pancreatic carcinoma (MIA Paca-2) in continuous culture: sensitivity to asparaginase. Int J Cancer 19:128–135. doi:10.1002/ijc.2910190118 832918

[33] Bressy C , Droby GN , Maldonado BD , Steuerwald N , Grdzelishvili VZ . 2019. Cell cycle arrest in G_2_/M phase enhances replication of interferon-sensitive cytoplasmic RNA viruses via inhibition of antiviral gene expression. J Virol 93:e01885-18. doi:10.1128/JVI.01885-18 30487274 PMC6364032

[34] Wennier ST , Liu J , Li S , Rahman MM , Mona M , McFadden G . 2012. Myxoma virus sensitizes cancer cells to gemcitabine and is an effective oncolytic virotherapeutic in models of disseminated pancreatic cancer. Mol Ther 20:759–768. doi:10.1038/mt.2011.293 22233582 PMC3321583

[35] Naqvi I , Gunaratne R , McDade JE , Moreno A , Rempel RE , Rouse DC , Herrera SG , Pisetsky DS , Lee J , White RR , Sullenger BA . 2018. Polymer-mediated inhibition of pro-invasive nucleic acid DAMPs and microvesicles limits pancreatic cancer metastasis. Mol Ther 26:1020–1031. doi:10.1016/j.ymthe.2018.02.018 29550075 PMC6079560

[36] Little EC , Wang C , Watson PM , Watson DK , Cole DJ , Camp ER . 2012. Novel immunocompetent murine models representing advanced local and metastatic pancreatic cancer. J Surg Res 176:359–366. doi:10.1016/j.jss.2011.10.025 22221605 PMC3406409

[37] Jazowiecka-Rakus J , Sochanik A , Hadryś A , Fidyk W , Chmielik E , Rahman MM , McFadden G . 2022. Combination of LIGHT (TNFSF14)-armed myxoma virus pre-loaded into ADSCs and gemcitabine in the treatment of experimental orthotopic murine pancreatic adenocarcinoma. Cancers (Basel) 14:2022. doi:10.3390/cancers14082022 35454928 PMC9027757

[38] Bian Z , Shi L , Kidder K , Zen K , Garnett-Benson C , Liu Y . 2021. Intratumoral SIRPα-deficient macrophages activate tumor antigen-specific cytotoxic T cells under radiotherapy. Nat Commun 12:3229. doi:10.1038/s41467-021-23442-z 34050181 PMC8163884

[39] Das M , Shen L , Liu Q , Goodwin TJ , Huang L . 2019. Nanoparticle delivery of RIG-I agonist enables effective and safe adjuvant therapy in pancreatic cancer. Mol Ther 27:507–517. doi:10.1016/j.ymthe.2018.11.012 30545600 PMC6401191

[40] Narayanan JSS , Ray P , Hayashi T , Whisenant TC , Vicente D , Carson DA , Miller AM , Schoenberger SP , White RR . 2019. Irreversible electroporation combined with checkpoint blockade and TLR7 stimulation induces antitumor immunity in a murine pancreatic cancer model. Cancer Immunol Res 7:1714–1726. doi:10.1158/2326-6066.CIR-19-0101 31409607 PMC6774877

[41] Her LS , Lund E , Dahlberg JE . 1997. Inhibition of ran guanosine triphosphatase-dependent nuclear transport by the matrix protein of vesicular stomatitis virus. Science 276:1845–1848. doi:10.1126/science.276.5320.1845 9188527

[42] Black BL , Rhodes RB , McKenzie M , Lyles DS . 1993. The role of vesicular Stomatitis virus matrix protein in inhibition of host-directed gene expression is genetically separable from its function in virus assembly. J Virol 67:4814–4821. doi:10.1128/JVI.67.8.4814-4821.1993 8392615 PMC237868

[43] Stojdl DF , Lichty BD , tenOever BR , Paterson JM , Power AT , Knowles S , Marius R , Reynard J , Poliquin L , Atkins H , Brown EG , Durbin RK , Durbin JE , Hiscott J , Bell JC . 2003. VSV strains with defects in their ability to shutdown innate immunity are potent systemic anti-cancer agents. Cancer Cell 4:263–275. doi:10.1016/s1535-6108(03)00241-1 14585354

[44] Cataldi M , Shah NR , Felt SA , Grdzelishvili VZ . 2015. Breaking resistance of pancreatic cancer cells to an attenuated vesicular stomatitis virus through a novel activity of IKK inhibitor TPCA-1. Virology 485:340–354. doi:10.1016/j.virol.2015.08.003 26331681 PMC4619123

[45] Weber H , Valenzuela D , Lujber G , Gubler M , Weissmann C . 1987. Single amino acid changes that render human IFN-alpha 2 biologically active on mouse cells. EMBO J 6:591–598. doi:10.1002/j.1460-2075.1987.tb04795.x 3034596 PMC553438

[46] van Pesch V , Lanaya H , Renauld J-C , Michiels T . 2004. Characterization of the murine alpha interferon gene family. J Virol 78:8219–8228. doi:10.1128/JVI.78.15.8219-8228.2004 15254193 PMC446145

[47] Felt SA , Droby GN , Grdzelishvili VZ , Lyles DS . 2017. Ruxolitinib and polycation combination treatment overcomes multiple mechanisms of resistance of pancreatic cancer cells to oncolytic vesicular stomatitis virus. J Virol 91:e00461-17. doi:10.1128/JVI.00461-17 28566376 PMC5533928

[48] Goad DW , Bressy C , Holbrook MC , Grdzelishvili VZ . 2022. Acquired chemoresistance can lead to increased resistance of pancreatic cancer cells to oncolytic vesicular stomatitis virus. Mol Ther Oncolytics 24:59–76. doi:10.1016/j.omto.2021.11.019 34977342 PMC8703189

[49] Amrutkar M , Gladhaug IP . 2017. Pancreatic cancer chemoresistance to gemcitabine. Cancers (Basel) 9:157. doi:10.3390/cancers9110157 29144412 PMC5704175

[50] Longley DB , Harkin DP , Johnston PG . 2003. 5-fluorouracil: mechanisms of action and clinical strategies. Nat Rev Cancer 3:330–338. doi:10.1038/nrc1074 12724731

[51] Wang Y , Zhang Y , Yang J , Ni X , Liu S , Li Z , Hodges SE , Fisher WE , Brunicardi FC , Gibbs RA , Gingras M-C , Li M . 2012. Genomic sequencing of key genes in mouse pancreatic cancer cells. Curr Mol Med 12:331–341. doi:10.2174/156652412799218868 22208613 PMC3799885

[52] Rhim JS . 1993. Neoplastic transformation of human cells in vitro. Crit Rev Oncog 4:313–335. 8485202

[53] Stewart BW , Haski R . 1984. Relationships between carcinogen metabolism, adduct binding and DNA damage in 3-methylcholanthrene-exposed lung. Chem Biol Interact 52:111–128. doi:10.1016/0009-2797(84)90087-5 6499078

[54] Crosby LM , Yoon LW , Easton MJ , Ni H , Morgan KT . 2010. Correction: transformation of Sv40-immortalized human uroepithelial cells by 3-methylcholanthrene increases IFN- and large T antigen-induced transcripts. Cancer Cell Int 10:10. doi:10.1186/1475-2867-10-10 20178601 PMC2848030

[55] Weichselbaum RR , Ishwaran H , Yoon T , Nuyten DSA , Baker SW , Khodarev N , Su AW , Shaikh AY , Roach P , Kreike B , Roizman B , Bergh J , Pawitan Y , van de Vijver MJ , Minn AJ . 2008. An interferon-related gene signature for DNA damage resistance is a predictive marker for chemotherapy and radiation for breast cancer. Proc Natl Acad Sci U S A 105:18490–18495. doi:10.1073/pnas.0809242105 19001271 PMC2587578

[56] Erdal E , Haider S , Rehwinkel J , Harris AL , McHugh PJ . 2017. A prosurvival DNA damage-induced cytoplasmic interferon response is mediated by end resection factors and is limited by Trex1. Genes Dev 31:353–369. doi:10.1101/gad.289769.116 28279982 PMC5358756

[57] Padariya M , Sznarkowska A , Kote S , Gómez-Herranz M , Mikac S , Pilch M , Alfaro J , Fahraeus R , Hupp T , Kalathiya U . 2021. Functional interfaces, biological pathways, and regulations of interferon-related DNA damage resistance signature (IRDS) genes. Biomolecules 11:622. doi:10.3390/biom11050622 33922087 PMC8143464

[58] Goad DW , Bressy C , Holbrook MC , Grdzelishvili VZ . 2022. Acquired chemoresistance can lead to increased resistance of pancreatic cancer cells to oncolytic vesicular stomatitis virus. Mol Ther Oncolytics 24:59–76. doi:10.1016/j.omto.2021.11.019 34977342 PMC8703189

[59] Noser JA , Mael AA , Sakuma R , Ohmine S , Marcato P , Lee PW , Ikeda Y . 2007. The RAS/Raf1/MEK/ERK signaling pathway facilitates VSV-mediated oncolysis: implication for the defective interferon response in cancer cells. Mol Ther 15:1531–1536. doi:10.1038/sj.mt.6300193 17505473

[60] Battcock SM , Collier TW , Zu D , Hirasawa K . 2006. Negative regulation of the alpha interferon-induced antiviral response by the Ras/Raf/MEK pathway. J Virol 80:4422–4430. doi:10.1128/JVI.80.9.4422-4430.2006 16611902 PMC1472035

[61] Christian SL , Zu D , Licursi M , Komatsu Y , Pongnopparat T , Codner DA , Hirasawa K . 2012. Suppression of IFN-induced transcription underlies IFN defects generated by activated Ras/MEK in human cancer cells. PLoS One 7:e44267. doi:10.1371/journal.pone.0044267 22970192 PMC3436881

[62] Yang L , Ding JL . 2019. MEK1/2 inhibitors unlock the constrained interferon response in macrophages through IRF1 signaling. Front Immunol 10:2020. doi:10.3389/fimmu.2019.02020 31507609 PMC6718554

[63] Deng H , Liu H , de Silva T , Xue Y , Mohamud Y , Ng CS , Qu J , Zhang J , Jia WWG , Lockwood WW , Luo H . 2019. Coxsackievirus type B3 is a potent oncolytic virus against KRAS-mutant lung adenocarcinoma. Mol Ther Oncolytics 14:266–278. doi:10.1016/j.omto.2019.07.003 31463367 PMC6709373

[64] Christian SL , Collier TW , Zu D , Licursi M , Hough CM , Hirasawa K . 2009. Activated Ras/MEK inhibits the antiviral response of alpha interferon by reducing STAT2 levels. J Virol 83:6717–6726. doi:10.1128/JVI.02213-08 19386709 PMC2698556

[65] Waters AM , Der CJ . 2018. KRAS: the critical driver and therapeutic target for pancreatic cancer. Cold Spring Harb Perspect Med 8:a031435. doi:10.1101/cshperspect.a031435 29229669 PMC5995645

[66] Erdal E , Haider S , Rehwinkel J , Harris AL , McHugh PJ . 2017. A prosurvival DNA damage-induced cytoplasmic interferon response is mediated by end resection factors and is limited by Trex1. Genes Dev 31:353–369. doi:10.1101/gad.289769.116 28279982 PMC5358756

[67] Luo J . 2021. KRAS mutation in pancreatic cancer. Semin Oncol 48:10–18. doi:10.1053/j.seminoncol.2021.02.003 33676749 PMC8380752

