# Supplementary material for: Intertumoral heterogeneity impacts oncolytic vesicular stomatitis virus efficacy in mouse pancreatic cancer cells
Source: J Virol. 2023 Sep 28;97(9):e01005-23. doi: 10.1128/jvi.01005-23 (PMC10537684; doi:10.1128/jvi.01005-23)
Supplement: Supplemental Figure S1 — Detailed karyotype analysis of mouse PDAC cell lines. [file jvi.01005-23-s0001.pdf]

Supplementary Figure S1. Spectral karyotyping (SKY) and multicolor fluorescence-in-situ-hybridization (mFISH) of mouse PDAC cell lines KPC-Luc-4580, KPC-Luc-A, and PANC02-Luc.

Each cell line was grown to 70% confluence in a T-75 flask. Adherent cells were harvested with colcemid arrest, treated with 0.75 M KCl hypotonic solution, and fixation with 3:1 methanol: acetic acid. The resulting cells were spread onto glass slides according to standard cytogenetic protocols. A Spectral Karyotyping (SKY) slide was processed according to the manufacturer's protocol (Applied Spectral Imaging). SKY uses a unique combination of five fluorescent dyes to paint all 24 chromosomes. G-band and SKY metaphase cells were imaged and karyotyped using Applied Spectral Imaging (ASI) software. Seven metaphase cells per sample were examined by SKY using the Olympus BX61 microscope with DAPI and SKY fluorescence filter sets. Karyotypes of all 7 cells are listed for each cell mouse PDAC cell line, while two representative karyograms are shown for two representative cells for each cell mouse PDAC cell line. The cytogenetic analyses were performed in the Cytogenomics Shared Resource at the University of Minnesota.

Cell Line: KPC-Luc-4580

CYTOGENETICS CORE LABORATORY

CLIENT: Dr. Valery Grdzelishvili (UNC Molecular Virology & Oncolytic Virotherapy Lab)

DATE RECEIVED: 10-18-2022

CELL LINE: 4580

METHODS: One T-250 flask of mouse pancreatic cancer cells was received on 10-18-22. Following a 4.0 hour colcemid treatment cells were harvested according to standard cytogenetic protocol. Seven metaphase cells were examined by multicolor fluorescence-in-situ-hybridization with Spectral Karyotyping (**SKY**).

RESULTS:

48~51,X,-Y,+1,+2,+3,+6,+8,+8,+11,+13,+15,+15,+18,+18,+19,+19,1~4ace[cp7]

INTERPRETATION:

All seven of the metaphase cells analyzed had a chromosome complement ranging from 48 (hyperdiploid) to 51 (hypotriploid). Based on a diploid karyotype in which there are two copies of each chromosome 1 through 19 and two sex chromosomes, all of these cells showed gains of an extra copy of chromosome 1 and two extra copies of chromosome 8, 15, 18 and 19. Also seen in the majority of cells were gains of chromosomes 2, 3, 6, 11 and 13. Unlike cell lines LUCA and PANC02, there were no clonal structural abnormalities with the exception of small marker chromosomes of unknown origin. Each of the seven cells examined had only one X chromosome present, no Y chromosome was detected. Without the germline material for comparison, it cannot be confirmed that the cells were derived from a male mouse.

Molly Lynch, CG(ASCP)
Cytogenetics Core Coordinator

B. Hirsch, PhD., FACMG
Cytogenetics Core Director

Cell Line: KPC-Luc-A

CYTOGENETICS CORE LABORATORY

CLIENT: Dr. Valery Grdzelishvili (UNC Molecular Virology & Oncolytic Virotherapy Lab)

DATE RECEIVED: 10-18-2022

CELL LINE: LUC A

METHODS: One T-250 flask of mouse pancreatic cancer cells was received on 10-18-22. Following a 4.0 hour colcemid treatment cells were harvested according to standard cytogenetic protocol. Seven metaphase cells were examined by multicolor fluorescence-in-situ-hybridization with Spectral Karyotyping (**SKY**).

RESULTS:
65~72,XX,-Y,-Y,+X,inv(X)(XA4XF3),+1,+2,-4,+5,del(5)(5C1),+8,+11,-12,+14,+15,
der(15)(15A1-->15F3::15?::15A1-->15F3),+17,+19,+19,2~3ace[cp7]

INTERPRETATION:
The seven metaphase cells analyzed by SKY had chromosome numbers ranging from 65 to 72, with five of the seven having a hypertriploid complement (65-70 chromosomes) and two having a near tetraploid complement (71, 72 chromosomes). Based on a true triploid karyotype (with three copies of each chromosome 1-19) there were gains of chromosomes 1, 2, 5, 8, 11, 14, 15, 17 and 19 in all cells along with various other combinations of chromosomal gains and losses that varied cell to cell. Structural abnormalities were also present including a deletion of 5q in 5 of 7 cells, an abnormality of 15 that resembled a duplication, and an inversion on an X chromosome in all cells. No Y chromosome was present in any of these 7 cells, with most having three X's, one of which had an inversion; without non-tumor tissue it cannot be confirmed that these tumor cells were derived from a male mouse.

Molly Lynch, CG(ASCP)
Cytogenetics Core Coordinator

B. Hirsch, PhD., FACMG
Cytogenetics Core Director

Cell Line: PANC02-Luc

CYTOGENETICS CORE LABORATORY

CLIENT: Dr. Valery Grdzelishvili (UNC Molecular Virology & Oncolytic Virotherapy Lab)

DATE RECEIVED: 10-18-2022

CELL LINE: PANC02

METHODS: One T-250 flask of mouse pancreatic cancer cells was received on 10-18-22. Following a 4.0 hour colcemid treatment cells were harvested according to standard cytogenetic protocol. Seven metaphase cells were examined by multicolor fluorescence-in-situ-hybridization with Spectral Karyotyping (**SKY**).

RESULTS:
77~90,XX,-Y,-Y,+X,+1,+3,der(4)t(4;2)(4E2;2?),+5,del(5)(11B5),-7,del(8)(8B2),-9,+10,+10,
+11,del(11)(11B5),+12,-13,+15,der(15)t(15;17)(15E2;17?),i(15)(15A1),+16,+17,+17,+19[cp7]

INTERPRETATION:
All seven of the metaphase cells analyzed by SKY had a near tetraploid complement, ranging from 77 to 90. Relative to a pure tetraploid complement (with four copies of each chromosome) there were gains of chromosomes 3 and 19 in all cells, as well as gains of chromosomes 10, 12 and 16 in most cells. As there was variability in the constellation of gains and losses between cells, the karyotype is described as a "composite karyotype." In addition to numerical abnormalities, there were a few structural abnormalities: a derivative chromosome comprised of chromosome 4 and 2 material in 5 of the 7 cells, a deletion of 5 in 3 of 7 cells, a deletion of 11 in 6 of 7 cells and a derivative chromosome comprised of chromosome 15 and 17 material. No Y chromosome was present in any of the 7 cells, with most of the cells having three X's; without non-tumor tissue it cannot be confirmed that these cells are derived from a male mouse.

Molly Lynch, CG(ASCP)
Cytogenetics Core Coordinator

B. Hirsch, PhD., FACMG
Cytogenetics Core Director

Seven metaphase cells per sample were examined by SKY using the Olympus BX61 microscope with DAPI and SKY fluorescence filter sets. Karyotypes of all 7 cells are listed for each cell mouse PDAC cell line

Cell Line: KPC-Luc-4580

4580	count	1	2	3	4	5	6	7	8	9	10	11	12	13	14	15	16	17	18	19	X	Y	
1	51	+1		+3		+5,+5,del(5F)	+6		+8,+8			+11		+13		+15	-16		+18	+19,+19		-Y	
2	49	+1	+2,+2	+3,+3		der(5;12)			+8,+8										+18,+18	+19		-Y	4ace
3	51	+1				+5	+6		+8			+11		+13		+15,+15			+18,+18	+19,+19		-Y	1ace
4	49	+1	+2	+3					+8							+15,+15			+18,+18	+19,+19		-Y	3ace
5	48	+1	+2						+8							+15			+18,+18	+19,+19		-Y	4ace
6	51	+1		+3			+6		+8,+8			+11		+13		+15	+16		+18	+19,+19		-Y	4ace
7	50	+1		+3			+6		+8,+8			+11		+13		der(15;19)			+18,+18	+19,+19		-Y	3ace

ISCN: 48~51,X,-Y,+1,+2,+3,+6,+8,+8,+11,+13,+15,+15,+18,+18,+19,+19,1~4ace[cp7]

Hyperdiploid karyotype (based on diploid 2N mouse karyotype)

Cell Line: KPC-Luc-A

LUC A	count	1	2	3	4	5	6	7	8	9	10	11	12	13	14	15	16	17	18	19	X	Y	
1	65	+1,+1	+2,+2			+5,del(5)	+6		+8,+8	+9	+10	+11,+11		+13	+14	+15,+15,der(15)	+16	+17,+17,der(17;X)	+18	+19,+19,+19	+X,inv(X)	-Y,-Y	
2	68	+1,+1,+1	+2,+2		del(4)	+5	+6,+6	+7,+7,+7	+8,+8,+8	+9,+9,+9		+11			+14,+14	+15,+15,der(15)	+16	+17,+17	+18	+19,+19	+X,inv(X)	-Y,-Y	
3	72	+1	+2	+3		+5,del(5)		+7	+8			+11	der(12;7)		+14	+15,der(15)		+17		+19,+19	+X,inv(X)	-Y,-Y	3ace
4	70	+1	+2		-4	+5,del(5)			+8,+8			+11	der(12;X)		+14	+15,der(15)		+17		+19,+19	+X,inv(X)	-Y,-Y	2ace
5	68	+1	+2		-4	+5,del(5)		i(7)	+8,+8			+11	-12			+15,der(15)		+17		+19,+19	+X,+X,inv(X)	-Y,-Y	3ace
6	71	+1	+2	+3	-4	+5			+8	+9		+11	-12		+14	+15,der(15)		+17		+19,+19	+X,inv(X)	-Y,-Y	2ace
7	68	+1	+2		-4	+5,del(5)			+8			+11	-12		+14	+15,der(15)		+17		+19,+19	+X,inv(X)	-Y,-Y	2ace

ISCN: 65~72,XX,-Y,-Y,+X,inv(X)(XA4XF3),+1,+2,-4,+5,del(5)(5C1),+8,+11,-12,+14,+15,der(15)(15A1-->15F3::15?::15A1-->15F3),+17,+19,+19,2~3ace[cp7]

Hypertriploid karyotype (based on triploid 3N mouse karyotype)

Cell Line: PANC02-Luc

PANC02	count	1	2	3	4	5	6	7	8	9	10	11	12	13	14	15	16	17	18	19	X	Y	
1	89	+1		+3		-5			+8,+8		+10		+12,+12,+12	-13	-14	+15	+16	+17,+17		+19	+X	-Y,-Y	
2	76		-2	+3,+3	der(4;2)		-6	-7		-9		del(11)	-12,-12	-13		der(15;7)	+16			+19,+19	+X	-Y,-Y	
3	88		+2	+3,+3	+4			-7		-9		del(11)	der(12;10)		-14	+15	+16,del(16)	+17,+17	+18,+18	+19		-Y,-Y	1ace
4	90	+1		+3	der(4;2)	+5,del(5)	+6,der(6;X)		del(8)		+10,+10	+11,del(11)	+12			der(15;17),i(15)	+16,der(16;19)	+17	i(18)	+19		-Y,-Y	
5	77	+1,+1,der(1)	-2	+3,+3	-4,der(4;2)	-5,del(5)	der(6;9)		-8	-9	-10	-11,del(11)	+12,+12	-13		-15,i(15)	-16	+17,+17	-18,-18	+19	-X,-X	-Y,-Y	
6	88			+3	der(4;2)	+5		-7	del(8)		+10,+10	+11,+11,del(11)	+12			+15,+15,der(15;17),i(15)	-16	+17,+17	-18	+19	+X	-Y,-Y	
7	90			+3	der(4;2)	+5,+5,del(5)		+7			+10,+10	+11,del(11)	+12			+15,der(15;17),i(15)	+16			+19	+X	-Y,-Y	

ISCN: 77~90,XX,-Y,-Y,+X,+1,+3,der(4)t(4;2)(4E2;2?),+5,del(5)(11B5),-7,del(8)(8B2),+10,+10,+11,del(11)(11B5),+12,+15,der(15)t(15;17)(15E2;17?),i(15)(15A1),+16,+17,+17,+19[cp7]

Hypertetraploid karyotype (based on tetraploid 4N mouse karyotype)

(yellow)	= SKY karyotype representative image
(green)	= clonal aberration
[cp#]	= composite karyotype, # of cells
ace	= acentric chromosome fragment
del(#)	= deletion
der(#;#)	= derivitive chromosome
inv(#)	= inversion/inverted chromosome
i(#)	- isochromosome

Seven metaphase cells per sample were examined by SKY using the Olympus BX61 microscope with DAPI and SKY fluorescence filter sets. Karyotypes of all 7 cells are listed for each cell mouse PDAC cell line

4580	ISCN **Cell Line: KPC-Luc-4580**
1	51,X,-Y,+1,+3,+5,+5,del(5)(5F)x2,+6,+8,+8,+11,+13,+15,-16,+18,+19,+19,2ace
2	49,X,-Y,+1,+2,+2,+3,+3,der(5)t(5;12)(5A1;12A1.2),+8,+8,+18,+18,+19,4ace
3	51,X,-Y,+1,+5,+6,+8,+11,+13,+15,+15,+18,+18,+19,+19,1ace
4	49,X,-Y,+1,+2,+3,+8,+15,+15,+18,+18,+19,+19,3ace
5	48,X,-Y,+1,+2,+8,+15,+18,+18,+19,+19,4ace
6	51,X,-Y,+1,+3,+6,+8,+8,+11,+13,+15,+16,+18,+19,+19,4ace
7	50,X,-Y,+1,+3,+6,+8,+8,+11,+13,der(15)t(15;19)(15A1;19A),+18,+18,+19,+19,3ace

LUC A	ISCN **Cell Line: KPC-Luc-A**
1	65,XX,-Y,-Y,+X,inv(X)(XA4XF3),+1,+2,-3,-4,del(5)(5C1),-7,+8,+11,-12,+15,der(15)(15A1-->15F3::15?::15A1-->15F3),+17,der(17)t(17;X),+19,+19,1ace
2	68,XX,-Y,-Y,+X,inv(X)(XA4XF3),+1,+1,+2,-3,-4,del(4)(4A5),+6,+7,+7,+8,+8,+9,+9,-10,-12,-13,+14,+15,der(15),+17,+19
3	72,XX,-Y,-Y,+X,inv(X)(XA4XF3),-4,del(5)(5C1),-6,-9,-10,-12,der(12)t(12;7)(12B3;7?),-13,der(15)(15A1-->15F3::15?::15A1-->15F3),-16,-18,+19,3ace
4	70,XX,-Y,-Y,+X,inv(X)(XA4XF3),-3,-4,-4,del(5),-6,-7,+8,-9,-10,der(12)t(12;X),-13,der(15),-16,-18,+19,2ace
5	68,XX,-Y,-Y,+X,+X,inv(X)(XA4XF3),-3,-4,-4,del(5),-6,-7,i(7),-9,-10,-12,-12,-13,-14,der(15),-16,-18,+19,3ace
6	71,XX,-Y,-Y,+X,inv(X)(XA4XF3),-4,-4,-6,-7,-10,-12,-12,-13,der(15),-16,-18,+19,2ace
7	68,XX,-Y,-Y,+X,inv(X)(XA4XF3),-3,-4,-4,del(5)(5C1),-6,-7,-9,-10,-12,-12,-13,der(15)(15A1-->15F3::15?::15A1-->15F3),-16,-18,+19,2ace

PANC02	ISCN **Cell Line: PANC02-Luc**
1	89,XX,-Y,-Y,+X,+1,+3,-5,+8,+8+10,+12,+12,+12,-13,-14,+15,+16,+17,+17,+19
2	76,XX,-Y,-Y,+X,-2,+3,+3,der(4)t(4;2)(4E2;2?),-6,-7,-9,del(11)(11B5),-12,12,-13,der(15)t(15;7)(15E2;7F3),+16,+19,+19
3	88,XX,-Y,-Y,+2,+3,-3,+4,-7,-9,del(11)(11B5),der(12)t(12;10)(12F2;10?),-14,+15,+16,del(16)(16B2),+17,+17,+18,+18,+19,+19,1ace
4	90,XX,-Y,-Y,+1,+1,+3,der(4;2)(4E2;2?),+5,del(5)(5C1),+6,der(6)t(6;X)(6B3;XB),del(8)(8B2),+10,+10,+11,del(11)(11B5),+12,+15,der(15)t(15;17)(15E2;17?),i(15)(15A1),+16,der(16)t(16;19)(16B5;19C1),+17,i(18)(18A1),+19
5	73,X,-X,-Y,-Y,+1,+1,der(1)t(1;8),-2,+3,-4,der(4;2),-5,del(5),der(6)t(6;9),-8,-9,-10,-11,del(11),+12,+12,-13,-15,i(15),-18,-18,+19
6	88,XX,-Y,-Y,+X,+3,der(4;2),+5,-7,del(8),+10,+10,+11,+11,del(11),+12,+15,+15,der(15;17),i(15),-16,+17,+17,-18,+19,+mar
7	90,XX,-Y,-Y,+X,+3,der(4;2)(4E2;2?),+5,+5,del(5)(5C1),+7,+10,+10,+11,del(11)(11B5),+12,+15,der(15;17)(15E2;17?),i(15)(15A1),+16,+16,+19

(yellow) = Representative SKY Karyotypes

Highlighted (representative) rows: KPC-Luc-4580 cells 4 and 6; KPC-Luc-A cells 3 and 7; PANC02-Luc cells 4 and 7.

University of Minnesota
Cytogenetics Laboratory Shared Resource

Cell Line: KPC-Luc-4580

Patient ID: 2022 UNC

Patient Name: 4580

Cell Results: 49,X, −Y, +1, +2, +3, +8, +15, +15, +18, +18, +19, +19,3ace

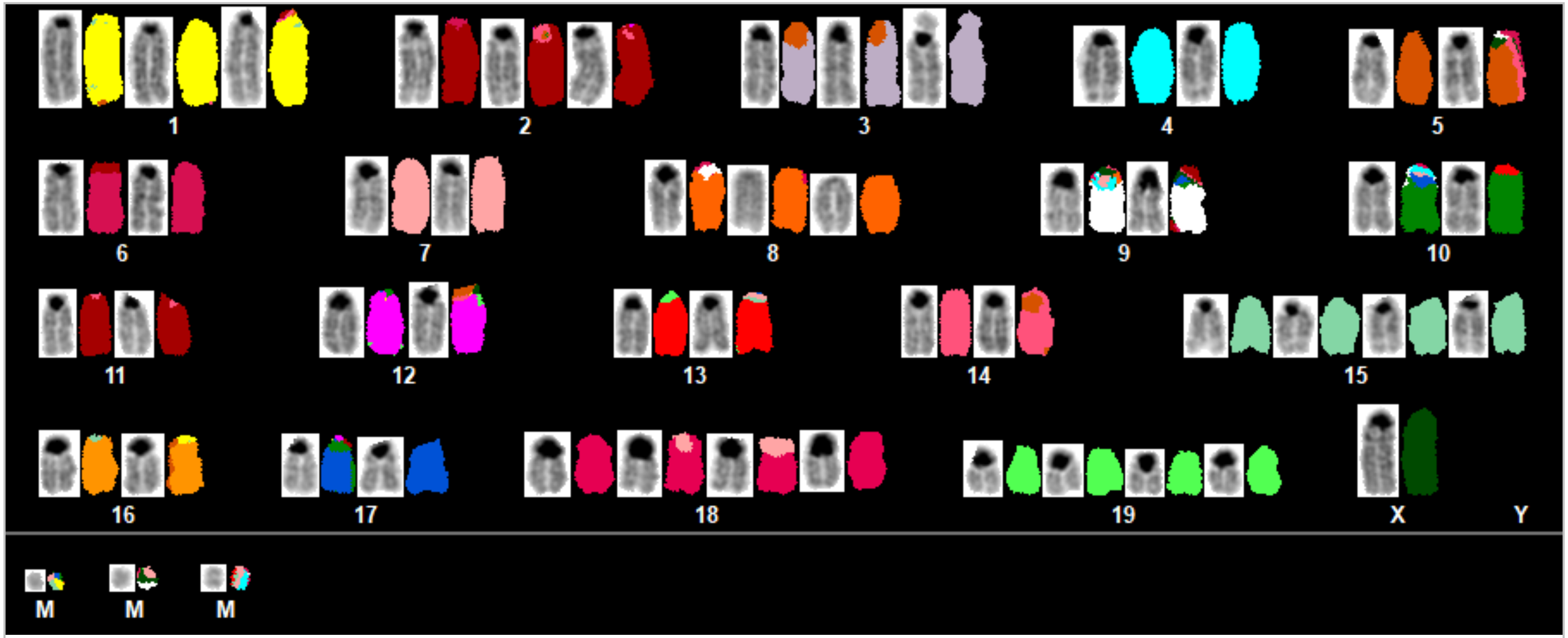

Label - Slide/Cell: 4580 - 2/14

University of Minnesota
Cytogenetics Laboratory Shared Resource

Cell Line: KPC-Luc-4580

Patient ID: 2022 UNC

Patient Name: 4580

Cell Results: 51,X, −Y, +1, +3, +6, +8, +8, +11, +13, +15, +16, +18, +19, +19,4ace

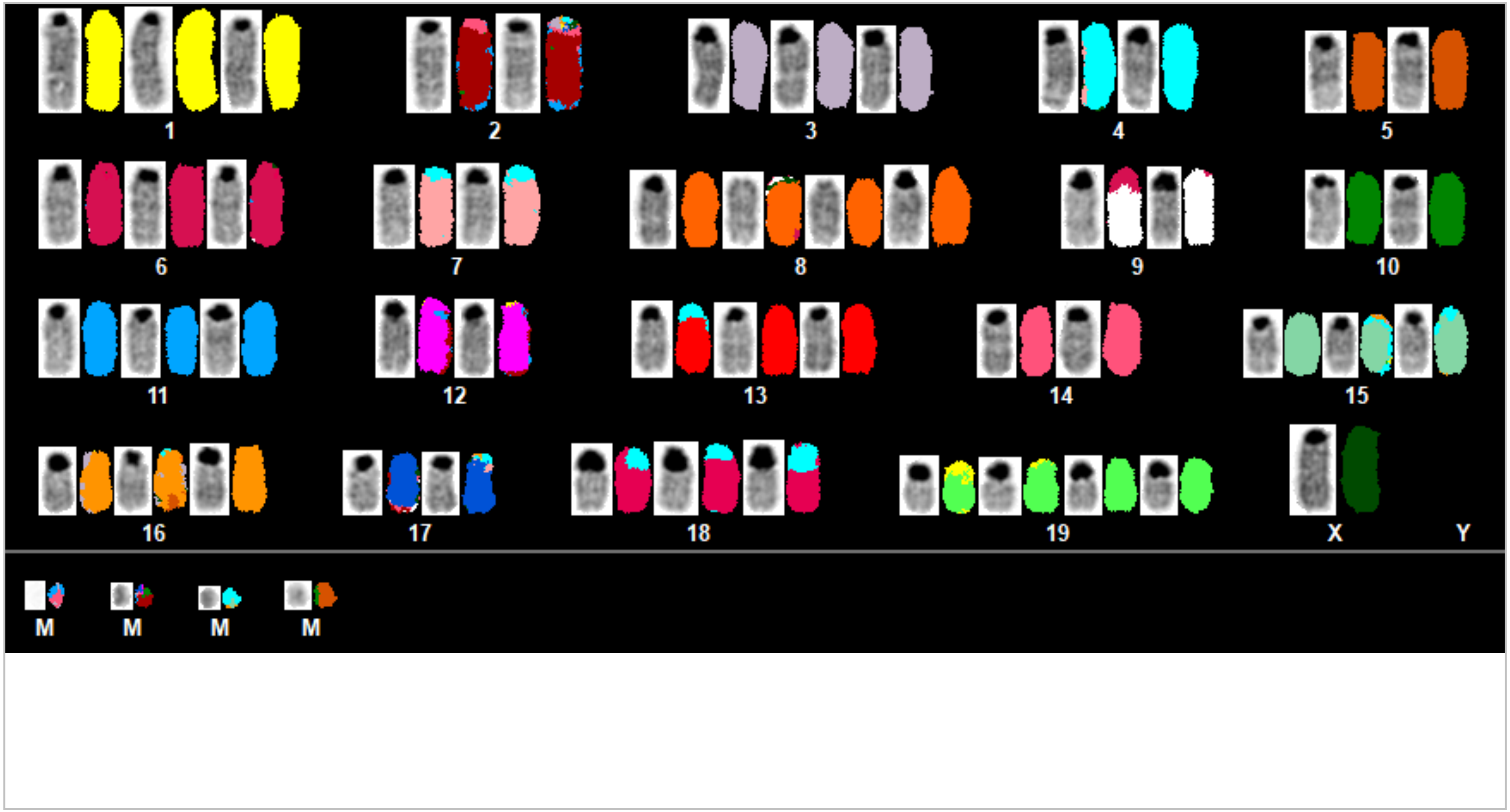

Label - Slide/Cell: 4580 - 2/18

X,Y:

University of Minnesota
Cytogenetics Laboratory Shared Resource

Cell Line: KPC-Luc-A

Patient ID: 2022 UNC

Patient Name: LUC A

Cell Results: 72,XX, −Y, −Y, +X,inv(X)(XA4XF3), +1, +2, +3, +5,del(5)(5C1), +7, +8, +11,der(12)t(12;7)(12B3;7?), +15, +15,der(15)(15A1-->15F3::15?::15A1-->15F3), +17, +19, +19,3ace

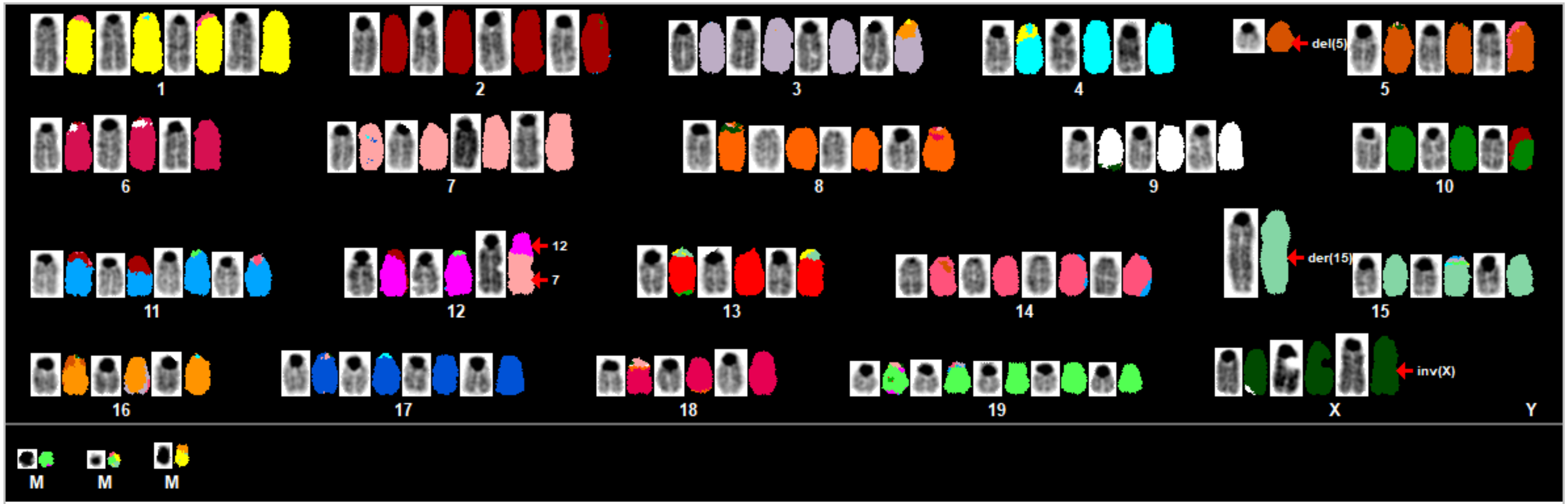

Label - Slide/Cell: LUC A - 4/3

Cell Line: KPC-Luc-A

Patient ID: 2022 UNC

Patient Name: LUC A

Cell Results: 68,XX, −Y, −Y, +X,inv(X)(XA4XF3), +1, +2, −4, +5,del(5)(5C1), +8, +11, −12, +14, +15,der(15)(15A1-->15F3::15?::15A1-->15F3), +17, +19, +19,2ace

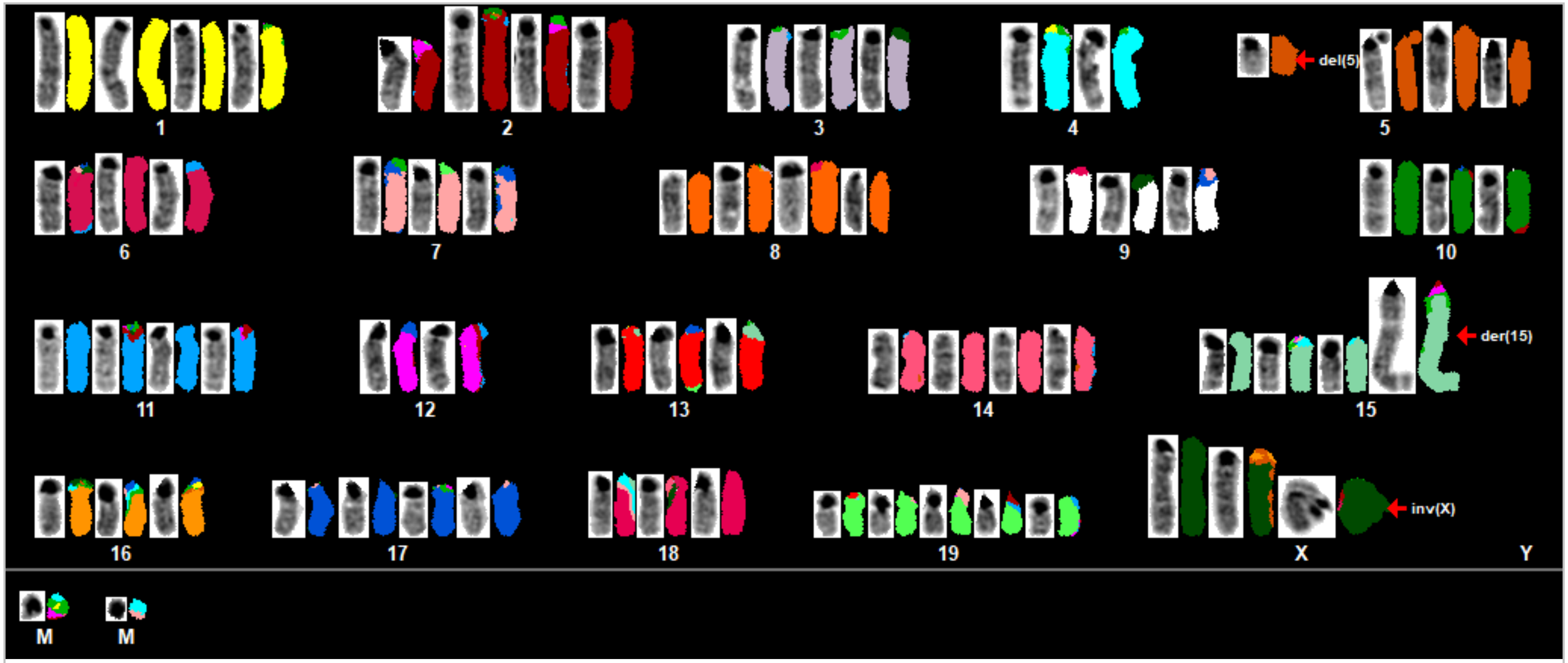

Label - Slide/Cell: LUC A - 4/12

UNIVERSITY OF MINNESOTA
Cytogenetics Laboratory Shared Resource

Case: 2022 UNC **Cell Line:** PANC02-Luc

Patient ID: 2022 UNC

Patient Name: PANC02

Cell Results: 90,XX, −Y, −Y, +1, +3,der(4;2)(4E2;2?), +5,del(5)(5C1), +6,der(6)t(6;X)(6B3;XB),del(8)(8B2), +10, +10, +11,del(11)(11B5), +12, +15,der(15)t(15;17)(15E2;17?),i(15)(15A1), +16,der(16)t(16;19)(16B5;19C1), +17,i(18)(18A1), +19

Preparation Date:

Analyzed by: lo

Cell Notes: Nonclonal der(6;X), der(16;19), i(18)

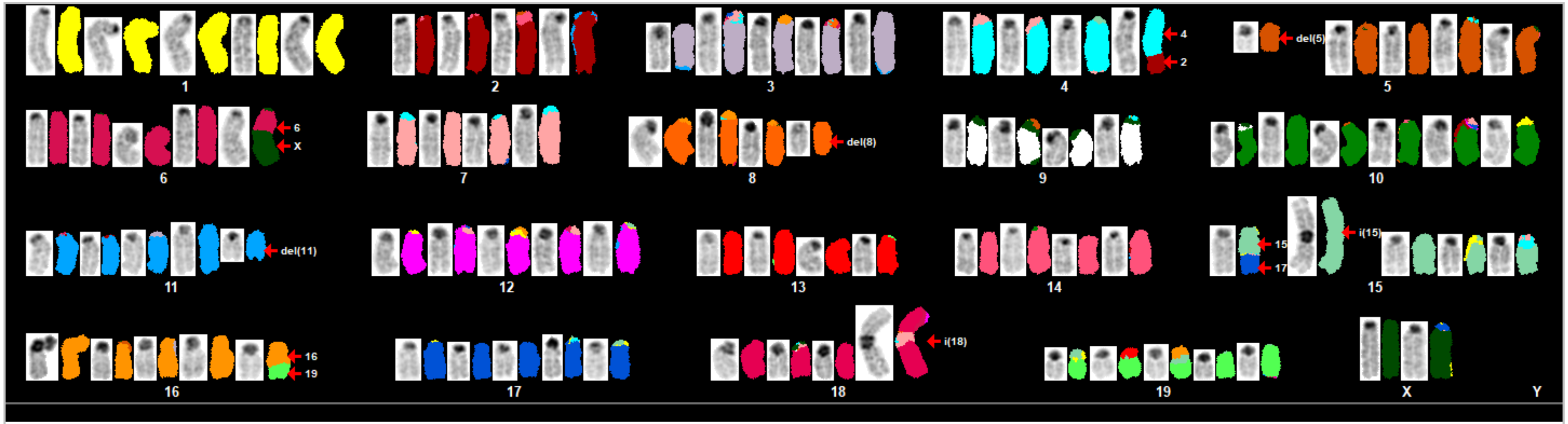

Report Date: Tuesday, January 10, 2023

Cell Line: PANC02-Luc

Patient ID: 2022 UNC

Patient Name: PANC02

Cell Results: 90,XX, −Y, −Y, +X, +3,der(4)t(4;2)(4E2;2?), +5, +5,del(5)(5C1), +7, +10, +10, +11,del(11)(11B5), +12, +15,der(15)t(15;17)(15E2;17?),i(15)(15A1), +16, +16, +19

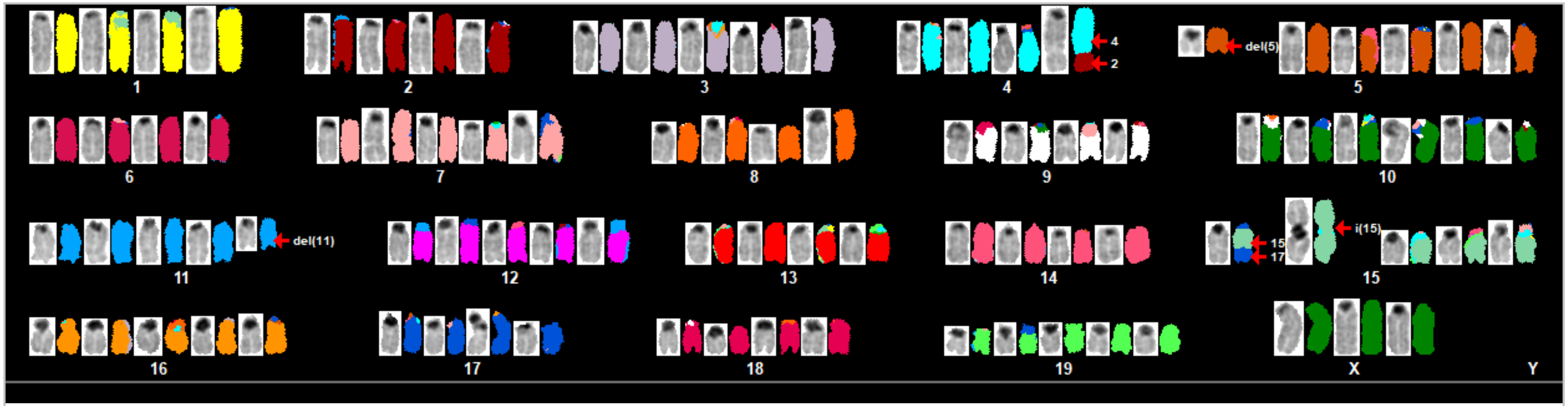

Label - Slide/Cell: PANC02 - 3/16
